# Transcriptional regulation of two redundant 3-bromo-4-hydroxybenzoate catabolic operons via two different regulatory modes in *Pigmentiphaga kullae* strain H8

**DOI:** 10.1128/aem.02403-24

**Published:** 2025-03-04

**Authors:** Zhuang Ke, Ke Yang, Zonghui Zhang, Ru Guo, Yuan Gao, Minjian Lan, Jiandong Jiang, Kai Chen

**Affiliations:** 1 Department of Microbiology, College of Life Sciences, Nanjing Agricultural University, Key Laboratory of Agricultural and Environmental Microbiology, Ministry of Agriculture and Rural Affairs 98430 , Nanjing, China; 2 College of Rural Revitalization, Jiangsu Open University 154504 , Nanjing, China; 3 Central Laboratory of College of Horticulture, Nanjing Agricultural University 70578 , Nanjing, China; Shanghai Jiao Tong University, Shanghai, China

**Keywords:** LTTR, MarR family, transcriptional regulation, 3-bromo-4-hydroxybenzoate, *Pigmentiphaga kullae*

## Abstract

Gene redundancy endows bacteria with enhanced adaptability to complex and fluctuating environments but results in genetic costs. Transcriptional regulation is considered an effective strategy for harmonizing adaptive benefits with physiological burdens. In our previous study, two redundant gene clusters (phbh1pcaApcaBorf404bhbR1 and phbh2pcaB2pcaA2bhbR2) involved in 3-bromo-4-hydroxybenzoate (3-Br-4-HB) catabolism were identified in *Pigmentiphaga kullae* strain H8. The LysR-type transcription regulator BhbR1, encoded by the bhbR1 gene, activated phbh1pcaApcaBorf404 transcription. Through DNase I footprinting assays, the presence of the inducer 3-Br-4-HB was found to shorten the BhbR1-bound region in the promoter, uncovering the protected -35 box, thereby activating transcription. The MarR-family transcription factor (MFTF) BhbR2, encoded by the bhbR2 gene, was different from typical inhibitive MFTFs and activated phbh2pcaB2pcaA2 transcription. BhbR2 was found to bind a 17-bp imperfect palindromic sequence (TTGATT-N5-AATCAA) in the target promoter. Intriguingly, the presence of 3-Br-4-HB neither dissociated BhbR2 from the promoter nor modified its binding site, indicating a novel regulatory mode. Despite a coincident trend in activating their respective operons in response to different concentrations of 3-Br-4-HB, BhbR1 and BhbR2 both showed a significant attenuation of the activation effect at high concentrations (>480 μM), highlighting the requisite co-existence of redundant 3-Br-4-HB catabolic operons and their regulatory genes. This study presents two distinct transcriptional regulation mechanisms of these two redundant 3-Br-4-HB catabolic operons in strain H8, expanding our understanding of the diversity of transcriptional regulation for enhancing adaptation.

**IMPORTANCE:**

In bacteria, catabolic genes for pollutant degradation often possess functionally redundant duplicates, providing a genetic basis for rapid adaptation to complex polluted environments. Synergic regulation plays an important role in balancing the physiological burden of extra genetic material with the adaptive benefits conferred by genetic redundancy. Although the co-existence of two redundant 3-bromo-4-hydroxybenzoate (3-Br-4-HB)-catabolic operons has been proven to enhance the metabolic robustness and adaptability of the host strain *Pigmentiphaga kullae* H8, how these two inducible catabolic operons are regulated remains unclear. This study identified two regulators, the LysR-type transcription regulator BhbR1 and the MarR-family transcription factor BhbR2, which activated transcription of the two 3-Br-4-HB-catabolic operons using different modes, and also revealed interactions of these two regulators with their effectors and target promoters. These findings not only clarify two distinct transcriptional strategies employed by redundant catabolic operons but also enhance our understanding of the significance of regulatory diversity for bacterial adaptation to complex polluted environments.

## INTRODUCTION

Bacteria play crucial roles in natural biogeochemical cycles, particularly in the degradation of environmental xenobiotics. They can metabolize various pollutants, including organohalogen compounds, polycyclic aromatic hydrocarbons, pesticides, plastics, and petroleum hydrocarbons, by expressing specific degrading enzymes (1–5). However, dynamic changes in the types and concentrations of pollutants in the environment necessitate effective strategies for regulating the expression of these genes. Transcriptional regulation serves as a fundamental mechanism to directly and effectively control gene expression (6, 7). It enables bacteria to quickly respond to specific compounds, control the expression of specialized catabolic genes, efficiently utilize these compounds as energy and carbon sources, and cope with nutritional requirements and toxicity pressures imposed by pollutants (8). Therefore, transcriptional regulation is critical for bacterial adaptation to complex and ever-changing polluted environments, broadening their ecological niches.

Genetic redundancy is a common occurrence across all three kingdoms of life (9). Intriguingly, species in highly disturbed habitats tend to have increased numbers of redundant genes compared to those in stable settings (10, 11). An increasing number of studies have indicated that genetic redundancy can confer hosts with adaptive benefits for coping with environmental disturbances, including buffering gene mutations, increasing protein dosage and function, and acquiring novel adaptive traits through neo- and sub-functionalization (9, 12–14). Despite its benefits, the expansion of genetic material imposes an additional physiological burden on the host. For instance, it consumes cellular resources and energy for gene replication, transcription, and translation, potentially compromising competitive fitness (15). Bacterial genomes often contain redundant genes for degrading pollutants, though their transcription varies in response to diverse environmental and physiological stimuli (16–19). Although experimental evidence is scarce, transcriptional regulation is intuitively believed to compensate for the physiological costs caused by genetic redundancy. Understanding the regulation mechanisms of these redundant genes that confer selective advantages and improve host adaptability to environmental disturbances has attracted widespread interest.

**Fig 1:**
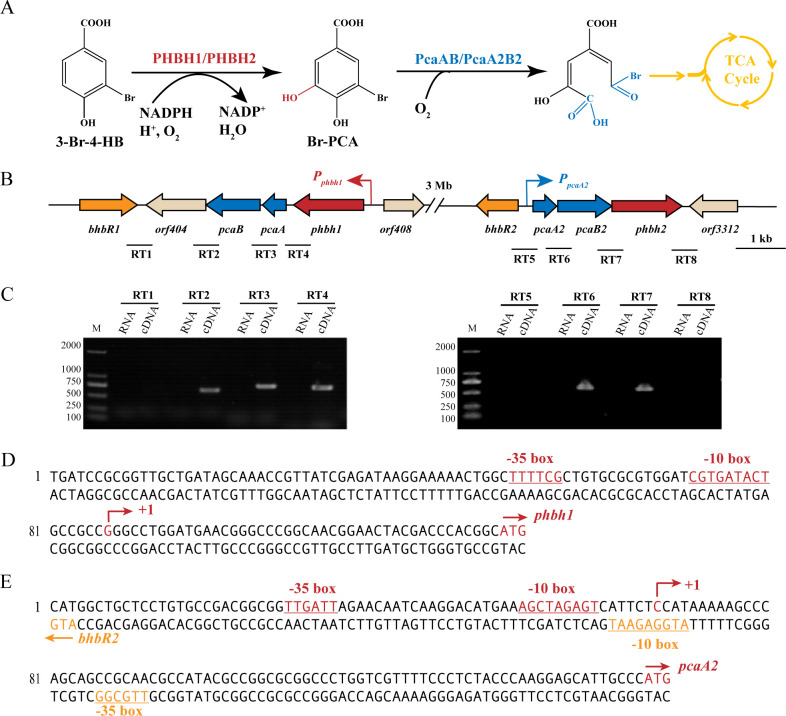
Organization and transcriptional analysis of two 3-Br-4-HB-degrading gene clusters (*bhb1* and *bhb2*) in *Pigmentiphaga kullae* strain H8. (**A**) Degradation pathway of 3-Br-4-HB. (**B**) Schematic diagram illustrating the genetic organization of the *bhb1* and *bhb2* clusters. Two promoters *P_phbh1_* and *P_pcaA2_*, corresponding to two operons *phbh1pcaApcaBorf404* and *phbh2pcaB2pcaA2,* are indicated by bent arrows. The amplification fragments (RT1-RT8) for transcriptional unit assessment are shown as black lines under the *bhb1* and *bhb2* clusters. (**C**) Transcriptional unit analysis of the *bhb1* and *bhb2* clusters. Total RNA and cDNA were used as templates for PCR amplification of eight fragments (RT1–RT8, as shown in panel B), which were then detected by electrophoresis. (**D**) Organization of the intergenic region between *orf408* and *pbhb1*. (**E**) Organization of the intergenic region between *bhbR2* and *pcaA2*. Start codons (ATG) are indicated by arrows, and the putative -10 boxes and -35 boxes are underlined. Transcriptional start sites are indicated by bent arrows, with the direction of the arrows indicating the direction of gene transcription.

In our previous study, *Pigmentiphaga kullae* strain H8, a bacterium capable of efficiently degrading 3-bromo-4-hydroxybenzoate (3-Br-4-HB), was isolated from soil highly contaminated with halogenated hydroxybenzoate (20, 21). Two redundant gene clusters, designated as *bhb1* (*phbh1pcaApcaBorf404bhbR1*) and *bhb2* (*phbh2pcaB2pcaA2bhbR2*), were confirmed to be involved in 3-Br-4-HB catabolism at both physiological and genetic levels (Fig. 1A and B). Notably, the redundancy of *bhb1* and *bhb2* clusters not only enables the host to metabolize anthropogenic 3-Br-4HB but also helps it overcome the toxicity and metabolic disturbance caused by high concentrations of 3-Br-4-HB, enhancing its adaptation to habitats highly contaminated with 3-Br-4-HB (21). The transcription of genes for key degradation enzymes, such as *phbh1* and *phbh2*, is inducible; however, their transcriptional regulation mechanisms remain unclear.

Here, a LysR-type transcriptional regulator BhbR1, encoded by a regulatory gene *bhbR1*, and a MarR-type transcriptional regulator BhbR2, encoded by a regulatory gene *bhbR2*, were found to be involved in the catabolism of 3-Br-4-HB in strain H8. The regulatory mechanisms of BhbR1 and BhbR2 for activating the transcription of two 3-Br-4-HB catabolic operons were analyzed at both physiological and genetic levels. This study will expand our understanding of the diversity of transcriptional regulation of redundant genes.

## RESULTS

### Transcriptional analysis of two 3-Br-4-HB-degrading gene clusters

Strain H8 harbors two spatially separate but functionally redundant 3-Br-4-HB-degrading gene clusters, *bhb1* and *bhb2*, located approximately 3 Mb apart on its genome (Fig. 1B). Reverse transcription PCR analysis of the intergenic regions between two adjacent open reading frames (*orfs*) was performed to delineate the transcriptional units of these clusters. Using the total RNA extracted from 3-Br-4-HB-induced cells of strain H8 and its reverse-transcribed cDNA as templates, three distinct segments, RT2, RT3, and RT4, were successfully amplified from the *bhb1* cluster (Fig. 1C), indicating that the *bhb1* cluster is organized into two operons: *phbh1pcaApcaBorf404* and *bhbR1*. Amplification of two segments (RT6 and RT7, Fig. 1C) from the *bhb2* cluster suggested that *bhb2* comprised two operons: *phbh2pcaB2pcaA2* and *bhbR2*.

To determine the promoter regions of 3-Br-4-HB-catabolic operons, *phbh1pcaApcaBorf404* and *phbh2pcaB2pcaA2*, transcriptional start sites (TSSs) were identified by 5′ rapid amplification of cDNA ends (5′-RACE) using total RNA extracted from 3-Br-4-HB-induced cells of strain H8. The sequencing results of 5′-RACE are shown in the supplemental material (Fig. S1). As shown in Fig. 1D and E, the TSSs were pinpointed at a G residue 44 bp upstream of the *phbh1* translational start codon and a C residue 79 bp upstream of the *pcaA2* translational start codon, respectively. The software BPROM (http://www.softberry.com/berry.phtml?topic=bprom&group=programs&subgroup=gfindb) was utilized to predict the -10 and -35 boxes of promoters. The predicted -10 (CGTGATAC) and -35 (TTTTCG) boxes of the *phbh1* promoter (designated *P_phbh1_*), and the predicted -10 (AGCTAGAGT) and -35 (TTGATT) boxes of the *pcaA2* promoter (designated *P_pcaA2_*) are shown in Fig. 1D and E, respectively.

We applied the RhoThermPredict algorithm to identify Rho-dependent terminators within the *bhb1* and *bhb2* gene clusters (22). In the *phbh1pcaApcaBorf404* operon*,* a Rho utilization site (RUT site) was predicted downstream of *orf404* (155–233 nt from the stop codon), followed by a PAUSE-CONSENSUS sequence (CAACGGTCTGC, 68–79 nt downstream of the RUT site) and a palindromic sequence (*GGCCGC*CGGG*GCGGCC*, nt 30–46) (italics denote a palindromic sequence) further downstream (Fig. S2A). Similarly, in the *phbh2pcaB2pcaA2* operon*,* a RUT site was predicted downstream of *phbh2* (+2 to −73 nt from the stop codon), accompanied by a PAUSE-CONSENSUS sequence (CGTCCGAGCCGA) and a palindromic sequence (*GCGGC*AGG*GCCGC*, nt 27–40) further downstream (Fig. S2B).

### BhbR1 and BhbR2 positively regulate the transcription of *phbh1pcaApcaBorf404* and *phbh2pcaB2pcaA2*, respectively

Two transcriptional regulatory genes, *bhbR1* and *bhbR2*, were found in the *bhb1* and *bhb2* clusters, respectively. BhbR1, encoded by the *bhbR1* gene and composed of 302 amino acids, showed the highest identity with the LysR-type transcriptional regulator (LTTR) TsaR (34.2%) from *Comamonas testosterone* (23). Phylogenetic analysis of BhbR1 and its closely related members showed that BhbR1, TsaR, GlaR, and TdcA formed a subclade from other LTTRs (Fig. S3A), indicating that BhbR1 is a new member of the LTTR family. BhbR2 is encoded by the *bhbR2* gene and consists of 179 amino acids; it was found to be most closely related to members of the MarR (multiple antibiotic resistance regulatory factor) family of transcriptional factors (MFTFs). Phylogenetic analysis revealed that BhbR2 is a new member of the MarR family, and the majority of MFTFs phylogenetically related to BhbR2 have been characterized as transcriptional repressors, including CbaR, EmrR, and HucR (Fig. S3B) (24–26).

**Fig 2:**
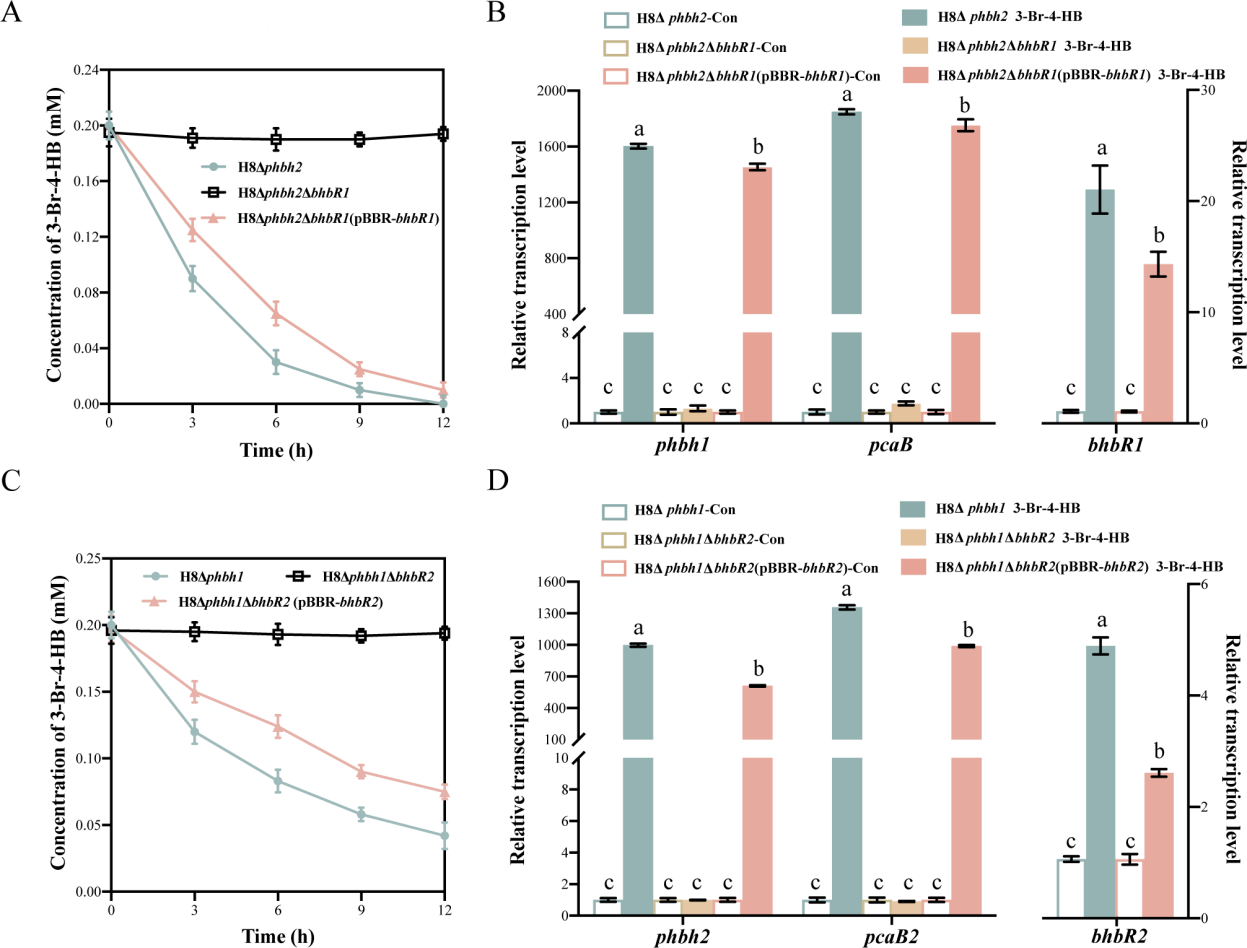
Roles of BhbR1 and BhbR2 in 3-Br-4-HB degradation and transcriptional regulation of degradation genes. (**A**) Degradation of 3-Br-4-HB by strain H8Δ*phbh2*, the *bhbR1*-mutant H8Δ*phbh2*Δ*bhbR1*, and the *bhbR1*-complemented strain H8Δ*phbh2*Δ*bhbR1* (pBBR-*bhbR1*). (**B**) Relative transcription analysis of *phbh1*, *pcaB*, and *bhbR1* in 3-Br-4-HB-induced and -uninduced cells of strains H8Δ*phbh2*, H8Δ*phbh2*Δ*bhbR1*, and H8Δ*phbh2*Δ*bhbR1* (pBBR-*bhbR1*). (**C**) Degradation of 3-Br-4-HB by strain H8Δ*phbh1*, the *bhbR2*-mutant H8Δ*phbh1*Δ*bhbR2*, and the *bhbR2*-complemented strain H8Δ*phbh1*Δ*bhbR2* (pBBR-*bhbR2*). (**D**) Relative transcription analysis of *phbh2*, *pcaB2*, and *bhbR2* in 3-Br-4-HB-induced and -uninduced cells of strains H8Δ*phbh1*, H8Δ*phbh1*Δ*bhbR2*, and H8Δ*phbh1*Δ*bhbR2* (pBBR-*bhbR2*). Error bars represent the standard deviations of three replicates.

Because both hydroxylases PHBH1 and PHBH2 catalyze the initial reaction of 3-Br-4-HB degradation in strain H8 (21), double-mutant strains, H8Δ*phbh2*Δ*bhbR1* and H8Δ*phbh1*Δ*bhbR2*, and their corresponding complemented strains, H8Δ*phbh2*Δ*bhbR1* (pBBR-*bhbR1*) and H8Δ*phbh1*Δ*bhbR2* (pBBR-*bhbR2*), were constructed to determine the transcriptional regulatory roles of BhbR1 and BhbR2. The degradation assay showed that the *bhbR1*-mutant strain H8Δ*phbh2*Δ*bhbR1* completely lost the capacity to degrade 3-Br-4-HB, whereas complementation with *bhbR1* in the mutant strain recovered this ability (Fig. 2A). Additionally, real-time quantitative PCR (RT-qPCR) analyses showed that in the presence of 3-Br-4-HB, transcription of target genes *phbh1* and *pcaB* was not activated in the mutant H8Δ*phbh2*Δ*bhbR1*; contrastingly, the transcription levels of *phbh1* and *pcaB* significantly increased in strain H8*Δphbh2* and the *bhbR1*-complemented strain H8Δ*phbh2*Δ*bhbR1* (pBBR-*bhbR1*) following 3-Br-4-HB induction (Fig. 2B). These results suggest that BhbR1 is involved in the catabolism of 3-Br-4-HB and activates the transcription of the *phbh1pcaApcaBorf404* operon in strain H8. Unlike typical LTTRs, BhbR1 also activates its own gene transcription, although to a lesser extent compared to the target genes (Fig. 2B).

Mutation of *bhbR2* in the strain H8Δ*phbh1*Δ*bhbR2* completely abolished 3-Br-4-HB degradation. The degradation ability was restored in the *bhbR2*-complemented strain H8Δ*phbh1*Δ*bhbR2* (pBBR-*bhbR2*), indicating the involvement of BhbR2 in the catabolism of 3-Br-4-HB in strain H8 (Fig. 2C). Furthermore, the *bhbR2*-mutant strain H8Δ*bhbR2*Δ*phbh1* did not upregulate transcription of the target genes *phbh2* and *pcaB2* with 3-Br-4-HB induction. However, significant transcriptional upregulation of the target genes was observed in the *bhbR2*-harboring strain H8Δ*phbh1* and the *bhbR2*-complemented strain H8Δ*phbh1*Δ*bhbR2* (pBBR-*bhbR2*) (Fig. 2D). These findings suggest that BhbR2 is an essential activator for the transcription of the *phbh2pcaA2pcaB2* operon in response to 3-Br-4-HB.

### Characterization of inducible effectors recognized by BhbR1 and BhbR2

**Fig 3:**
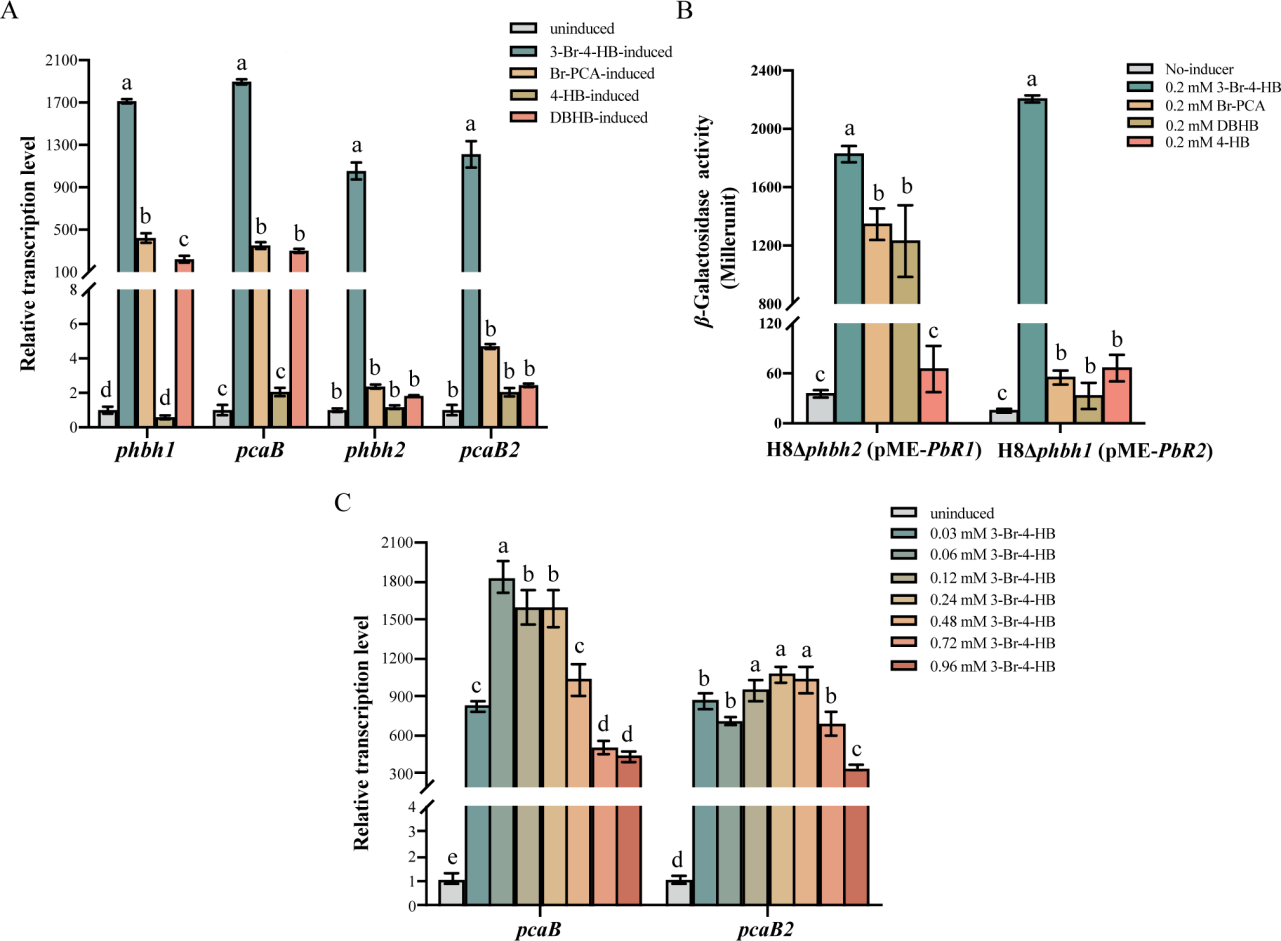
Identification of inducers recognized by BhbR1 and BhbR2 and the effect of 3-Br-4-HB concentration on target gene transcription. (**A**) Transcription analysis of *phbh1* and *pcaB* in strain H8Δ*phbh2*, and *phbh2* and *pcaB2* in strain H8Δ*phbh1*, was conducted in the presence or absence of 3-Br-4-HB, Br-PCA, 4-HB, or DBHB. (**B**) The *β*-galactosidase activity assay was conducted in strains H8Δ*phbh2* (pME-*PbR1*) and H8Δ*phbh1* (pME-*PbR2*) upon induction with 0.2 mM of 3-Br-4-HB, Br-PCA, DBHB, and 4-HB. (**C**) Transcription analysis of target genes *pcaB* and *pcaB2* in strain H8Δ*phbh1*Δ*phbh2* was performed in response to varying concentrations of 3-Br-4-HB (0, 30, 60, 120, 240, 480, 720, and 960 µM). Error bars represent the standard deviations of three replicates. Different letters indicate differences as determined by one-way ANOVA (*P* < 0.05).

To identify the inducers that activate target gene transcription by BhbR1 and BhbR2, we analyzed the transcription levels of *phbh1*, *pcaB*, *phbh2*, and *pcaB2* in the presence of 3-Br-4-HB, the intermediate metabolite 3-bromo-4,5-dihydroxybenzoate (Br-PCA), and the structural analogs 3,5-dibromo-4-hydroxybenzoate (DBHB) and 4-hydroxybenzoate (4-HB) by RT-qPCR. As shown in Fig. 3A, transcription levels of four target genes were significantly upregulated under 3-Br-4-HB induction, indicating that 3-Br-4-HB is an inducer for both BhbR1 and BhbR2. In addition, transcription of *phbh1* and *pcaB* showed moderate upregulation relative to the control when induced with Br-PCA and DBHB, suggesting that BhbR1 has non-specific inducer recognition. The presence of 4-HB did not trigger any notable transcriptional response.

The *β*-galactosidase activity assay was conducted to further consolidate the recognition of inducers by BhbR1 and BhbR2. Strains H8Δ*phbh2* (pME-*PbR1*) and H8Δ*phbh1* (pME-Pb*R2*) were induced with 0.2 mM concentrations of 3-Br-4-HB, Br-PCA, DBHB, and 4-HB, respectively. As shown in Fig. 3B, for strain H8Δ*phbh2* (pME-*PbR1*), *β*-galactosidase activity increased 51.7-fold with 3-Br-4-HB, 38.2-fold with Br-PCA, and 34.9-fold with DBHB, relative to non-induced controls. In contrast, *β*-galactosidase activity in H8Δ*phbh1* (pME-*PbR2*) was significantly increased only with 3-Br-4-HB as an inducer, with a 145.6-fold increase over non-induced cells. These results align with RT-qPCR data, further demonstrating that 3-Br-4-HB is a shared inducer for both BhbR1 and BhbR2, while BhbR1 also recognizes Br-PCA and DBHB.

To investigate the effect of inducer concentration on the activity of regulators, the transcriptional differences of target genes in the two operons induced by different concentrations of 3-Br-4-HB (0, 30, 60, 120, 240, 480, 720, and 960 µM) were further analyzed. Simultaneously, to avoid the fluctuation of inducer concentration caused by the degradation of 3-Br-4-HB, transcriptional analysis of target genes *pcaB* and *pcaB2* was performed using the double mutant strain H8*∆phbh1∆phbh2* that had complete loss of 3-Br-4-HB-degrading ability. As shown in Fig. 3C, *pcaB* and *pcaB2* exhibited the strongest transcriptional activity at inducer concentrations ranging from 60 to 240 µM and 30 to 480 µM, respectively. However, there was a significant decrease in transcriptional activity for both *pcaB* and *pcaB2* as the inducer concentration increased; when the inducer concentration reached 960 µM, the transcription of *pcaB* and *pcaB2* was only upregulated by 438- and 348-fold, respectively. These data suggest that high concentrations of 3-Br-4-HB induce physiological toxicity, affecting gene transcription.

### Identification of the binding sites of promoter regions by BhbR1 and BhbR2

The C-terminal His-tagged BhbR1 and BhbR2 were over-expressed in *Escherichia coli* BL21 (DE3), purified using Ni-NTA affinity chromatography, and verified by SDS-PAGE (Fig. S4). Gel filtration analysis revealed that active BhbR1 exists as a tetramer in PBS (8.0 g NaCl, 0.2 g KCl, 1.5 g Na_2_HPO_4_, 0.24 g KH_2_PO_4_, and 1 L deionized water, pH 7.8) (Fig. S5B), whereas BhbR2 forms a dimer (Fig. S5D).

**Fig 4:**
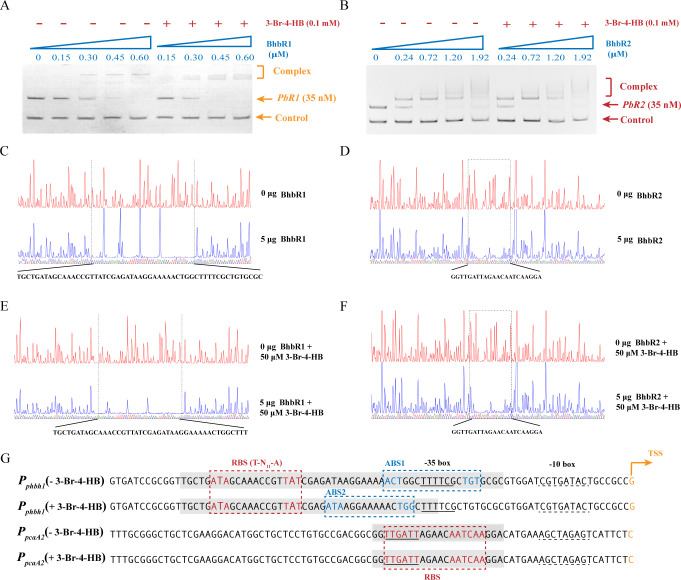
Identification of the promoter binding sites of BhbR1 and BhbR2. (**A**) EMSA of BhbR1 binding with the promoter DNA probe *PbR1* (**A**) and BhbR2 binding with the promoter DNA probe *PbR2* (**B**). “−” indicates samples incubated without 3-Br-4-HB, while “+” indicates samples incubated with 0.1 mM 3-Br-4-HB. Each lane contained 35 nM of promoter DNA probe. A 152-bp DNA fragment amplified from the 16S rRNA gene of strain H8 was used as a control DNA probe (28 nM). Protein-DNA complexes are indicated by brackets. DNase I footprinting assays of BhbR1 binding with the probe *PbR1* in the absence (**C**) or presence (**E**) of 50 µM 3-Br-4-HB, and BhbR2 binding with the probe *PbR2* in the absence (**D**) or presence (**F**) of 50 µM 3-Br-4-HB. The 6-carboxyfluorescein (FAM)-labeled DNA probe (350 ng) was incubated with 5 µg of BhbR1 or BhbR2, indicated by blue lines, as well as incubated without BhbR1 or BhbR2, indicated by red lines. Regions protected by BhbR1 or BhbR2 from DNase I cleavage are indicated with black dotted boxes, and the sequences of protected regions are shown at the bottom. (**G**) DNA elements in the promoters *P_phbh1_* and *P_pcaA2_*. The promoter regions protected by BhbR1 and BhbR2 in the absence (−) or presence (+) of 3-Br-4-HB are marked with light gray stripes. The regulatory binding sites for BhbR1 (ATA-N_9_-TAT) and BhbR2 (TTGATT-N_5_-AATCAA) are framed with red dashed lines. Two putative low-affinity activation binding sites (ABS1 and ABS2) in the promoter *P_phbh1_* are framed with blue dashed lines. The putative -10 and -35 boxes are underlined by black dashed and solid lines, respectively. TSSs are indicated by orange bent arrows, with the direction of the arrows indicating the direction of gene transcription.

Electrophoretic mobility shift assays (EMSAs) were performed to determine the interactions between the regulators (BhbR1 and BhbR2) and their target promoter DNA probes: *PbR1* (a 243-bp DNA fragment containing the promoter *P_phbh1_*) and *PbR2* (a 253-bp DNA fragment containing the promoter *P_pcaA2_*). As shown in Fig. 4A and B, regardless of the presence of the inducer 3-Br-4-HB, both BhbR1 and BhbR2 bound to their corresponding target promoter probes and formed protein-DNA complexes, BhbR1-*PbR1* and BhbR2-*PbR2*, delaying the migration of the two promoter probes. In contrast, nonspecific DNA probe *Pb16S*, a 152-bp 16S rRNA gene fragment from strain H8, showed no interaction with either regulator; this confirmed the specificity of BhbR1 and BhbR2 for their target regions of *P_phbh1_* and *P_pcaA2_*, respectively.

DNase I footprint assays were carried out to identify the detailed BhnR1- and BhbR2-binding sites in their target promoter regions. In the absence of the inducer, BhbR1 protected a 54-bp DNA sequence (5′-TGCTGATAGCAAACCGTTATCGAGATAAGGAAAAACTGGCTTTTCGCTGTGCGC-3′), located from -22 to -75 relative to the TSS of the promoter *P_phbh1_* (Fig. 4C). This protected region contains a high-affinity regulatory binding site (RBS), a 15-bp imperfect palindromic sequence (ATA-N_9_-TAT) with a consensus T-N_11_-A motif of a typical LTTR RBS, usually centered near position -65 in LTTR promoter regions (8, 27), and a putative low-affinity activation binding site (ABS1) ACT-N_10_-TGT overlapping the -35 box (Fig. 4G). The presence of 3-Br-4-HB shortened the BhbR1-protected region, located from -33 to -75 relative to the TSS of *P_phbh1_* (Fig. 4E). This suggests that the activation binding site of BhbR1 was shifted to a position upstream of ABS1, designated as ABS2 (Fig. 4G).

BhbR2 consistently shielded a 22-bp invariant sequence (5′-TCCTTGATTGTTCTAATCAACC-3′) spanning from -22 to -43 in relation to the TSS of promoter *P_pcaA2_*, regardless of the presence of 3-Br-4-HB (Fig. 4D and F), which was consistent with the results of EMSA. The protected region covered the -35 box of *P_pcaA2_* and included an AT-rich non-continuous palindromic sequence (TTGATTGTTCTAATCAA), which was consistent with the characteristics of typical MFTFs.

### Determination of regulator binding dynamics with the inducer and the target promoter

**Fig 5:**
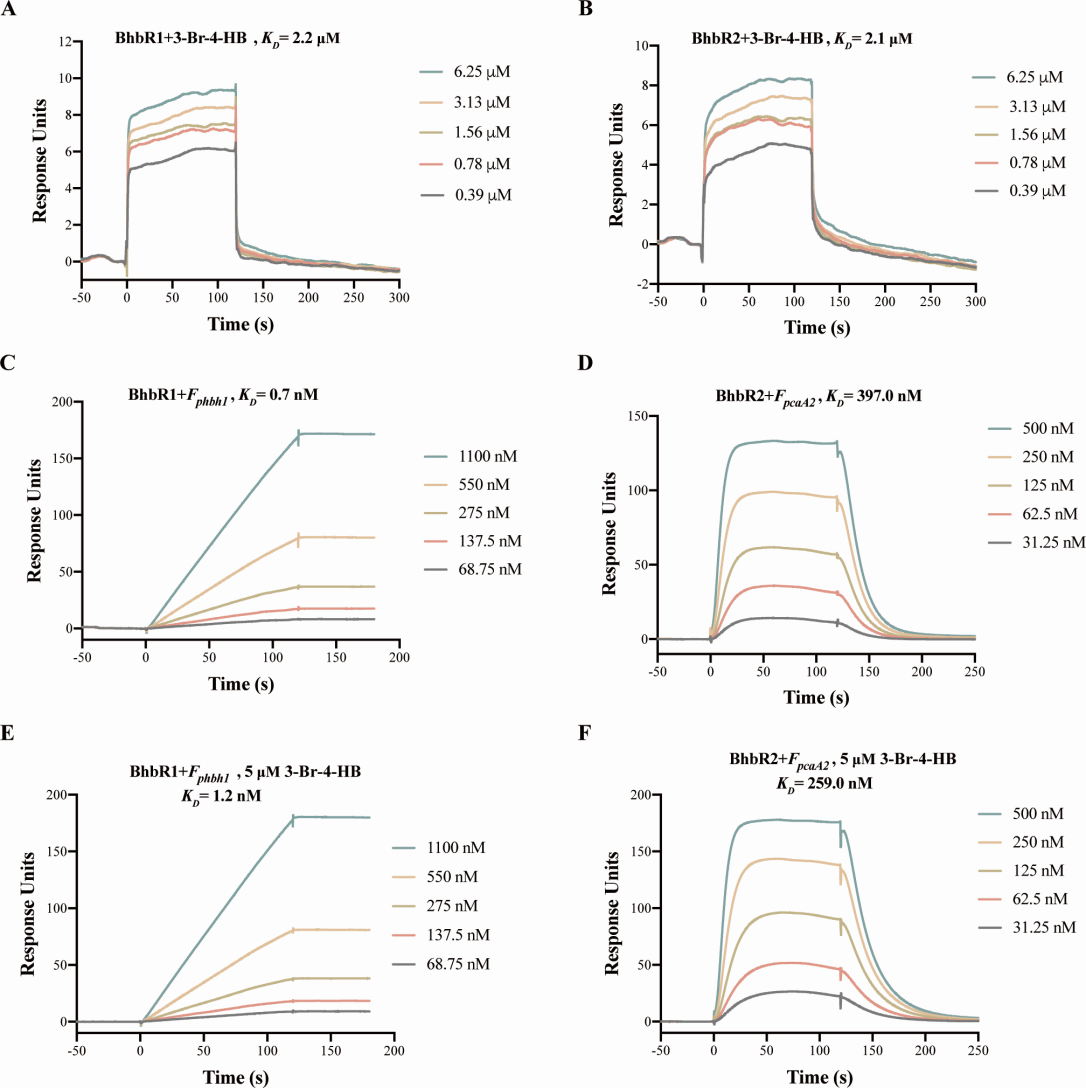
SPR assays for binding dynamics of the regulators with the inducer and target promoters. SPR assays analyzing interactions of the inducer 3-Br-4-HB with regulators BhbR1 (**A**) and BhbR2 (**B**). A serial dilution of 3-Br-4-HB (0.39–6.25 µM) was flowed over the CM7 sensor chip. SPR assays of BhbR1 binding with the fragments *F_phbh1_* in the absence (**C**) or presence (**E**) of 5 µM 3-Br-4-HB, and BhbR2 binding with the fragments *F_pcaA2_* in the absence (**D**) or presence (**F**) of 5 µM 3-Br-4-HB. Biotinylated fragments *F_phbh1_* and *F_pcaA2_* were captured on an SA sensor chip, while nonspecific biotinylated fragments *F_16S_*, derived from the 16S rRNA gene of strain H8, served as a control. Various concentrations of BhbR1 (68.75–1,100 nM) and BhbR2 (31.25–500 nM) were passed over the immobilized DNA fragments. The sensor surface was regenerated after each cycle using a short treatment with 0.5% (wt/vol) SDS. Derived dissociation constants (*K_D_*) are displayed above the equilibrium binding curves.

Surface plasmon resonance (SPR) assays were performed to evaluate the binding dynamics between the regulators BhbR1 and BhbR2 and the inducer 3-Br-4-HB, as well as the promoters *P_phbh1_* and *P_pcaA2_*, respectively. As shown in Fig. 5A and B, the equilibrium dissociation constants (*K_D_*) for the binding of BhbR1 and BhbR2 with 3-Br-4-HB were 2.2 and 2.1 µM, respectively, indicating comparable affinity. BhbR1 displayed a strong affinity (*K_D_* = 0.7 nM) toward the biotinylated promoter fragment *F_pbhb1_*, whereas BhbR2 exhibited a low affinity (*K_D_* = 397.0 nM) for the biotinylated promoter fragment *F_pcaA2_* (Fig. 5C and D). However, upon the addition of 3-Br-4-HB, the affinity of BhbR1 for *F_phbh1_* decreased, as indicated by an increased *K_D_* value of 1.20 nM, whereas the affinity of BhbR2 for *F_pcaA2_* was enhanced, resulting in a reduced *K_D_* value of 259.0 nM (Fig. 5E and F). These results indicate that the addition of 3-Br-4-HB subtly modulates the binding and dissociation thresholds of BhbR1 and BhbR2 with their respective target promoters.

## DISCUSSION

3-Br-4-HB is both naturally occurring as well as a synthetic compound widely used as an intermediate in chemical synthesis (1, 28, 29). Although the microbial degradation of 3-Br-4-HB has been extensively studied at the genetic and biochemical levels (1, 30), the regulatory mechanisms remain unclear. In this study, we demonstrated that the LysR-type transcriptional regulator BhbR1 and the MarR-family transcription factor BhbR2 trigger the transcription of two redundant 3-Br-4-HB-degrading gene clusters in response to 3-Br-4-HB, thereby governing 3-Br-4-HB metabolism in strain H8. This research not only fills a gap in the regulatory mechanisms of 3-Br-4-HB microbial metabolism but also deepens our understanding of the transcriptional regulation of redundant genes in microorganisms.

LTTRs are the most abundant and extensively studied transcriptional regulators in bacteria, managing various physiological and metabolic processes by activating or repressing target gene transcription (31–33). In the regulation of aromatic catabolism, LTTRs typically activate the transcription of catabolic genes while repressing their own gene transcription (8). In contrast, BhbR1 not only strongly activates target operon transcription but also slightly activates its own gene transcription (Fig. 2B). Structurally, LTTRs contain a highly conserved winged-helix-turn-helix (wHTH) DNA-binding domain at the N-terminus and an effector-binding domain at the C-terminus (34). Physiologically active LTTRs typically form homotetramers composed of two dimers, each binding to a high-affinity RBS and a low-affinity ABS on the target promoter, respectively (35, 36). DNase I footprinting assays revealed that the addition of 3-Br-4-HB shortened the BhbR1-binding region, allowing a BhbR1 dimer to slide from ABS1 to ABS2 (Fig. 4E and G), thereby exposing the -35 box and initiating transcriptional activation. This interaction between BhbR1 and the target promoter resembles the proposed “sliding dimer” model of LTTRs: the conformational contraction induced by effectors affects the entire protein-DNA complex, leading to the relaxation of the DNA bending and exposure of the -35 box, which facilitates the recruitment of RNA polymerase to activate transcription (27, 33, 37, 38).

MFTFs are widely distributed in bacteria and archaea and play important roles in various biological processes, including antibiotic resistance, stress responses, virulence, and the catabolism of aromatic compounds (39–43). Typically, MFTFs consist of 148–196 amino acids and feature a wHTH DNA-binding motif and a ligand-binding site, enabling conformational changes in response to specific signals (8, 44). Most MFTFs are homodimers, with each wHTH binding to an inverted repeat sequence within a palindromic sequence (16–20 bp) in the promoter region (45). The overlap of the binding sites of MFTFs with the -10 and -35 boxes leads to transcriptional repression of target genes; upon binding effectors, MFTFs undergo a conformational change, causing dissociation from the promoter DNA and relieving transcriptional repression (45, 46). To date, most MFTFs have been identified as transcriptional repressors, with only a few acting as activators (41, 47, 48). In this study, transcriptional activation of the *phbh2pcaA2pcaB2* operon was observed exclusively in the presence of *bhbR2* (Fig. 2C and D), suggesting that BhbR2 functions as a transcriptional activator in the catabolism of 3-Br-4-HB. Notably, the MFTFs with the closest phylogenetic relationship to BhbR2 in the phylogenetic tree have been identified as transcriptional repressors (Fig. S3B), highlighting the unique role of BhbR2. Furthermore, EMSA and DNase I footprinting assays showed that the addition of the inducer 3-Br-4-HB did not dissociate BhbR2 from the promoter DNA or alter its promoter-binding site (Fig. 4F). Collectively, these findings suggest a novel regulatory mechanism by which BhbR2 positively regulates the transcription of the *phbh2pcaA2pc*aB2 operon, although further investigation is needed to fully understand this process.

Regulating the transcription of redundant genes in response to specific signals is an effective strategy for balancing selective advantages and physiological burdens. A classic example was found in *Pseudomonas putida* mt-2, where the transcription of the redundant catechol 1,2-dioxygenase gene *catA2* was selectively triggered in response to intracellular excess catechol, functioning as a “metabolic safety valve” to alleviate the toxicity of excessive catechol (49). In strain H8, the redundancy of the two 3-Br-4-HB-catabolic operons has been confirmed to help the host cope with toxic stress and metabolic disturbances caused by high concentrations of 3-Br-4-HB (21). When exposed to varying concentrations of 3-Br-4-HB, BhbR1 and BhbR2 consistently activated transcription of their respective operons, with no selective transcription favoring either operon in response to high or low concentrations of the inducer (Fig. 3C). This observation was further supported by the similar *K_D_* values of BhbR1 and BhbR2 with 3-Br-4-HB (Fig. 5A and B). Nevertheless, high concentrations of 3-Br-4-HB (0.72 and 0.96 mM) adversely affected strain H8 cells, resulting in a significant attenuation of the transcriptional activation mediated by BhbR1 and BhbR2 (Fig. 3C). Therefore, equipping redundant 3-Br-4-HB-catabolic operons with two distinct regulatory systems is an effective safeguard measure to ensure the production of sufficient degradation enzymes, enabling host cells to cope with the toxic effects of high concentrations of 3-Br-4-HB in the habitat.

## MATERIALS AND METHODS

### Chemicals, bacterial strains, culture medium, plasmids, and oligonucleotides

All chemical standards (purities ≥ 98%), 3-Br-4-HB, Br-PCA, DBHB, and 4-HB, were purchased from Bidepharm (Shanghai, China) or J&K Chemical Co., Ltd. (Shanghai, China). Molecular biology reagents, such as restriction enzymes, high-fidelity DNA polymerase, PrimeScript RT reagent Kit with gDNA Eraser, TB Green Premix Ex Taq II (Tli RNaseH Plus) Kit, and BCA Protein Assay Kit, were purchased from TakaRa Biotechnology Co., Ltd. (Dalian, China). ClonExpress II One Step cloning kit was purchased from Vazyme Biotech Co., Ltd. (Nanjing, China).

The strains and plasmids used in this study are summarized in Table 1, and oligonucleotide primers are listed in Table 2. The method and results of phylogenetic analysis of *Pigmentiphaga kullae* strain H8 are shown in Text S1. Lysogenic broth (LB) medium (10.0 g tryptone, 5.0 g yeast extract, 5.0 g NaCl, and 1 L deionized water) was used to cultivate *E. coli* strains and *Pigmentiphaga kullae* strains in culture tubes or Erlenmeyer flasks with shaking (180 rpm) at 37°C. Mineral salts medium (MSM; 1.0 g NH_4_Cl, 1.5 g K_2_HPO_4_, 0.5 g KH_2_PO_4_, 0.2 g MgSO_4_, 1.0 g NaCl, and 1 L deionized water, pH 7.0) supplemented with 0.2 mM 3-Br-4-HB was used for degradation assays. YMS medium (MSM supplemented with 0.2% [wt/vol] yeast extract) was used to prepare substrate-induced cells. The solid medium was prepared by adding 2.0% (wt/vol) agar to the liquid medium. Antibiotic concentrations were used as follows: kanamycin at 50 mg L^−1^, spectinomycin at 80 mg L^−1^, and ampicillin and gentamicin at 100 mg L^−1^.

**TABLE 1:** Strains and plasmids used in this study

Strains and plasmids	Relevant characteristics*^a^*	Source or reference
*Pigmentiphaga kullae* strains		
H8	3-Br-4-HB-degrading strain, wild type; Spe^r^, Str^r^	(21)
H8Δ*phbh1*	Mutant of strain H8 with *phbh1* knockout; Spe^r^, Str^r^	(21)
H8Δ*phbh2*	Mutant of strain H8 with *phbh2* knockout; Spe^r^, Str^r^	(21)
H8Δ*phbh2*Δ*bhbR1*	Double mutant of strain H8 with *phbh2* and *bhbR1* knockout; Spe^r^, Str^r^	This study
H8Δ*phbh1*Δ*bhbR2*	Double mutant of strain H8 with *phbh1* and *bhbR2* knockout; Spe^r^, Str^r^	This study
H8Δ*phbh2*Δ*bhbR1* (pBBR-*bhbR1*)	Mutant H8Δ*phbh2*Δ*bhbR1* complemented with *bhbR1* gene by harboring the plasmid pBBR-*bhbR1*; Spe^r^, Str^r^, Km^r^	This study
H8Δ*phbh1*Δ*bhbR2* (pBBR-*bhbR2*)	Mutant H8Δ*phbh1*Δ*bhbR2* complemented with *bhbR2* gene by harboring the plasmid pBBR-*bhbR2*; Spe^r^, Str^r^, Km^r^	This study
H8Δ*phbh1*Δ*phbh2*	Double mutant of strain H8 with *phbh1* and *phbh2* knockout; Spe^r^, Str^r^	(21)
H8Δ*phbh2* (pME-*PbR1*)	Mutant H8Δ*phbh2* harboring the plasmid pME-*PbR1*; Spe^r^, Str^r^, Tc^r^	This study
H8Δ*phbh1* (pME-*PbR2*)	Mutant H8Δ*phbh1* harboring the plasmid pME-*PbR2*; Spe^r^, Str^r^, Tc^r^	This study
*E. coli* strains		
DH5α	Host strain for cloning vectors	Lab stored
HB101 (pRK2013)	Conjugation helper strain; Km^r^	Lab stored
BL21(DE3)	Host strain for expression vectors	Lab stored
Plasmids		
pBBR1MCS-2	Broad host range vector; Km^r^	(50)
pBBR-*bhbR1*	The *bhbR1* gene cloned into pBBR1MCS-2; Km^r^	This study
pBBR-*bhbR2*	The *bhbR2* gene cloned into pBBR1MCS-2; Km^r^	This study
pJQ200SK	Suicide vector; P15A, *ori, sacB,* RP4, (pBluescriptSK); Gm^r^	Lab stored
pJQ-Δ*bhbR1*	pJQ200SK containing the upstream and downstream fragments of *bhbR1* gene for the gene targeting of *bhbR1* gene; Gm^r^	This study
pJQ-Δ*bhbR2*	pJQ200SK containing the upstream and downstream fragments of *bhbR2* gene for the gene targeting of *bhbR2* gene; Gm^r^	This study
pET29a(+)	Expression vector; Km^r^	Lab stored
pET-*bhbR1*	The *bhbR1* gene cloned into pET29a(+); Km^r^	This study
pET-*bhbR2*	The *bhbR2* gene cloned into pET29a(+); Km^r^	This study
pMD19-T	TA clone vector; Amp^r^	TaKaRa
pMD-*PbR1*	pMD19-T harboring the fragment of DNA probe *PbR1*; Amp^r^	This study
pMD-*PbR2*	pMD19-T harboring the fragment of DNA probe *PbR2*; Amp^r^	This study
pME6522	Vector for promoter-*lacZ* transcriptional fusions; Tc^r^	(35)
pME-*PbR1*	pME6522 harboring the DNA fragment *PbR1* containing the promoter *P_phbh1_*; Tc^r^	This study
pME-*PbR2*	pME6522 harboring the DNA fragment *PbR2* containing the promoter *P_pcaA2_*; Tc^r^	This study

^*a*^ Spe^r^, spectinomycin resistance; Km^r^, kanamycin resistance; Gm^r^, gentamicin resistance; Amp^r^, ampicillin resistance; Str^r^, streptomycin resistance; and Tc^r^, tetracycline resistance.

**TABLE 2:** Primers used in this study

Primers	Sequence (5′−3′)*^a^*	Purpose
Gene knockout, complementation, and overexpression		
bhbR1-kn1 bhbR1-kn2	CAATCTAGACTGCTGGCCAACATGGTGGA (XbaI) GTCTGCAACAGGGGCGTGCTGCAGTTCGACCTTGGGGAAG	Amplification of the upstream homologous arm of *bhbR1* for gene knockout
bhbR1-kn3 bhbR1-kn4	CTTCCCCAAGGTCGAACTGCAGCACGCCCCTGTTGCAGAC CAACTCGAGTGTGGGTGCACAAGGACACG (XhoI)	Amplification of the downstream homologous arm of *bhbR1* for gene knockout
bhbR1-f bhbR1-r	GTAGAATTCATCATGGCGGCTTCGACAA (EcoRI) GAAGGTACCAACCGCGGGCCCTTCTA (KpnI)	Amplification of *bhbR1* for gene complementation
bhbR1-29f bhbR1-29r	GCACATATGACGCTGCAGCAACTGCGCG (NdeI) GCACTCGAGCCGGCCCATGCCGGCGAT (Xho I)	Amplification of *bhbR1* gene for cloning into pET29a(+) vector
bhbR2-kn1 bhbR2-kn2	CAATCTAGAGTGCAGGAGCTGATACAG (XbaI) CCTTCCGAACTGGCCGAACGGGAAAGAATCCGGGAGCT	Amplification of the upstream homologous arm of *bhbR2* for gene knockout
bhbR2-kn3 bhbR2-kn4	CGAGCTCCCGGATTCTTTCCCGTTCGGCCAGTTCGGAA CAAGGGCCCGTCAGACCGCTCCCTTGT (ApaI)	Amplification of the downstream homologous arm of *bhbR2* for gene knockout
bhbR2-f bhbR2-r	GCAGGTACCTTGGGTAGAGGGAAAA (KpnI) CAAGGATCCTACCAGACGCCCGCCAGGT (BamHI)	Amplification of *bhbR2* for gene complementation
bhbR2-29f bhbR2-29r	GCACATATGTCCTTCGAGCAGCCCGCAA (NdeI) GCACTCGAGCCAGACGCCCGCCAGGTC (XhoI)	Amplification of *bhbR2* gene for cloning into pET29a(+) vector
Transcriptional organization		
RT-F1 RT-R1	TGGGCGTGGTGGCCCGCAAGG CTTCCCGAGGTGCCCACGCTC	Amplification of 724 bp of *bhbR1-orf404*-spanning region
RT-F2 RT-R2	CACCTGGTTGAAGGTGGCCAC TCGTCGGCACCGGCGGCA	Amplification of 685 bp of *orf404-pcaB*-spanning region
RT-F3 RT-R3	CAGCGGCACGACCTTGATGTT GGGCATAGACGGCACCTATGT	Amplification of 780 bp of *pcaB-pcaA-spanning* region
RT-F4 RT-R4	TAGAGCCCGGCTCCCACCGTC CCTGAACGAAGGCCGCATCGT	Amplification of 734 bp of *pcaA-phbh1-*spanning region
RT-F5 RT-R5	CGTTCGGCCAGTTCGGAA CAGCTTGAGCAGGAAGTA	Amplification of 592 bp of *bhbR2-pcaA2*-spanning region
RT-F6 RT-R6	ACGAAGAGGCCTACATGA CGTCGTAGACCAGCGATT	Amplification of 556 bp of *pcaA2-pcaB2-*spanning region
RT-F7 RT-R7	GCAAGATGGACCAGGACT CACTTCATGCTGGGGATA	Amplification of 553 bp of *pcaB2-phbh2*-spanning region
RT-F8 RT-R8	GGTGATGAAGACCTACGA CCAATTGCGCAAGGTGTT	Amplification of 757 bp of *phbh2-orf3312*-spanning region
RT-qPCR		
Qphbh1-f Qphbh1-r	CATGGTCCACGAAGGCATA CAGGTCCTTGACGATTTCA	Amplification of a 121-bp fragment of *phbh1* for RT-qPCR
QpcaB-f QpcaB-r	CATAGGCAGTTCGCACGTT GTACTTCGTTCTCCAGCCA	Amplification of a 125-bp fragment of *pcaB* for RT-qPCR
Qphbh2-f Qphbh2-r	CGGTGATGAAGACCTACGA GAAGCTCAGCATGGCGAAG	Amplification of a 125-bp fragment of *phbh2* for RT-qPCR
QpcaB2-f QpcaB2-r	ACCTGGAATTCTCGCAGCA GTATGGTCGAAGCACAGGT	Amplification of a 124-bp fragment of *pcaB2* for RT-qPCR
QbhbR1-f QbhbR1-r	GGTCATGGCGCTGGTCAAT AGGCAGATCTCGAAGCTGT	Amplification of a 132-bp fragment of *bhbR1* for RT-qPCR
QbhbR2-f QbhbR2-r	GTTCAGCATGTCGTCGAGC CATCGAACTGACCGACAAG	Amplification of a 127-bp fragment of *bhbR2* for RT-qPCR
Q16S-f Q16S-r	GTTCTTCGGAGCTTGGTAG ACATCATCCACCGCTTGTG	Amplification of a 115-bp fragment of 16S rRNA gene for RT-qPCR
5′-RACE		
phbh1-GSP1 phbh1-GSP2 phbh1-GSP3	GTTTCGCCTTCCTGCTCGAAGC CGATTTCATGCTGGGGATAGA CGAAGCGTATGTCTATGCCTT	5′-RACE of the operon *phbh1pcaApcaBorf404*
pcaA2-GSP1 pcaA2-GSP2 pcaA2-GSP3	CGTGGTCGTTGTAGACCATGAT ATGGAGGGCACGTGAGAAGTA GTCAGACCGCTCCCTTGTCGT	5′-RACE of the operon *pcaA2pcaB2phbh2*
EMSA and DNase I footprinting assays		
PbR1-f PbR1-r	GAACTCGAGGTGCTGTCGATTTGTGCCA (XhoI) GAAACTAGTCGTGGGTCGTAGTTCCGT (SpeI)	Amplification of DNA probe *PbR1* (243 bp) containing the promoter *P_phbh1_* for EMSA
PbR2-f PbR2-r	GAACTCGAGTGATGCCGTAGCGGCCTT (XhoI) GAAACTAGTGGGCAATGCTCCTTGGGTA (SpeI)	Amplification of DNA probe *PbR2* (258 bp) containing the promoter *P_pcaA2_* for EMSA
Pb16S-f Pb16S-r	GCGGCGAGTGGCGAACGGGTGAG TAATCCGACATCGGCCGCTCCAA	Amplification of a nonspecific probe *Pb16S* (152 bp) from 16S rRNA gene of strain H8 as control for EMSA
FAM-M13F M13R	GTAAAACGACGGCCAGT (5′-FAM labeled) CAGGAAACAGCTATGAC	Amplification of DNA probes for DNase I footprinting assays
SPR assay		
Fpb1-Btn-f Fpb1-r	GTCGTAGTTCCGTTGCCGGG (5′-biotin labeled) ACCAGCAAGGAACGTGCCCC	Amplification of biotinylated DNA fragments *F_pbhb1_* (144 bp) for SPR
Fpb2-f Fpb2-Btn-r	CGAAGGACATGGCTGCTCCT GGGCAATGCTCCTTGGGTAG (5′-biotin labeled)	Amplification of biotinylated DNA fragments *F_pcaA2_* (153 bp) for SPR
F16S-Btn-f F16S-r	GCGGCGAGTGGCGAACGGGTGAG (5′-biotin labeled) TAATCCGACATCGGCCGCTCCAA	Amplification of nonspecific biotinylated DNA fragments *F_16S_* (152 bp) for SPR
*β*-galactosidase activity assay		
pME-PbR1-F	ATCGGAATTCGTGCTGTCGATTTGTGCCA (EcoRI)	Amplification of the DNA fragment *PbR1* (243 bp) containing the promoter *P_pbhb1_* for constructing pME-*PbR1*
pME-PbR1-R	ATCGCTGCAGCGTGGGTCGTAGTTCCGT (PstI)	
pME-PbR2-F	ATCGGAATTCTGATGCCGTAGCGGCCTT (EcoRI)	Amplification of the DNA fragment *PbR2* (258 bp) containing the promoter *P_pcaA2_* for constructing pME-*PbR2*
pME-PbR2-R	ATCGCTGCAGGGGCAATGCTCCTTGGGTA (PstI)	

^*a*^ The restriction enzyme sites are underlined.

### Detection of TSSs of the *phbh1pcaApcaBorf404* and *phbh2pcaB2pcaA2* operons

The TSSs of the *phbh1pcaApcaBorf404* and *phbh2pcaB2pcaA2* operons were identified using a 5′/3′-RACE Kit (2nd Generation, Roche, Switzerland) following the manufacturer’s instructions. Briefly, total RNA was extracted from strain H8 cells induced by 0.2 mM 3-Br-4-HB, using TRIzol reagent (Invitrogen). First-strand cDNA was synthesized with a primer (phbh1-GSP1 or pcaA2-GSP1) by reverse transcription. The cDNA was incubated with terminal transferase and dATP for homopolymeric tailing. Tailed cDNA was amplified using an oligo dT-anchor primer from the kit and a specific primer (phbh1-GSP2 or pcaA2-GSP2). The obtained PCR product was used as a template for nested PCR with the oligo dT-anchor primer and a specific primer (phbh1-GSP3 or pcaA2-GSP3), and the resulting product was cloned into pMD19-T (TaKaRa) for sequencing.

### Gene disruption and complementation

The upstream and downstream homologous arms of *bhbR1* and *bhbR2* were amplified with primer pairs, bhbR1-Kn1/Kn2 with bhbR1-Kn3/Kn4, and bhbR2-Kn1/Kn2 with bhbR2-Kn3/Kn4, respectively. The upstream and downstream fragments of both *bhbR1* and *bhbR2* were fused by overlapping PCR and inserted into pJQ200SK to generate *bhbR1*-disrupted plasmid pJQ-Δ*bhbR1* and *bhbR2*-disrupted plasmid pJQ-Δ*bhbR2*. Then, pJQ-Δ*bhbR1* and pJQ-Δ*bhbR2* were transferred into strains H8Δ*phbh2* and H8Δ*phbh1*, respectively, by triparental mating with the helper strain *E. coli* HB101 (pRK2013). The *bhbR1*-disrupted mutant H8Δ*phbh2*Δ*bhbR1* and *bhbR2*-disrupted mutant H8Δ*phbh1*Δ*bhbR2* were obtained using a method that has been previously described (21).

The PCR-amplified sequences of *bhbR1* and *bhbR2* were individually cloned into the broad-host-range vector pBBR1MCS-2 using a ClonExpress II one-step cloning kit (Vazyme, Nanjing, China). The resulting constructs (pBBR-*bhbR1* and pBBR-*bhbR2*) were transformed into mutants H8Δ*phbh2*Δ*bhbR1* and H8Δ*phbh1*Δ*bhbR2*, respectively, using triparental mating. The *bhbR1-*complemented strain H8Δ*phbh2*Δ*bhbR1* (pBBR-bhbR1) and the *bhbR2-*complemented strain H8Δ*phbh2*Δ*bhbR2* (pBBR-bhbR2) were then used for further experiments.

### RT-qPCR

Cells of various *Pigmentiphaga kullae* strains were grown in LB broth overnight, harvested by centrifugation, and washed twice with fresh YMS medium. Cells were then inoculated into YMS medium at an OD_600_ value of 0.4 and incubated with 0.2 mM 3-Br-4-HB, Br-PCA, or DBHB for 1.5 h or 0.2 mM 4-HB for 0.5 h to prepare substrate-induced cells. Cells were grown in YMS medium without additives for 1.5 h to generate uninduced control cells. The extraction of total RNA from induced and uninduced cells, generation of cDNA, and RT-qPCR were then carried out according to the previously described methods (32). Relative transcription of target genes was quantified using the 2^-ΔΔ*CT*^ threshold cycle (*C_T_*) method (51), and the 16S rRNA gene of strain H8 was used as a reference for normalization. The Δ*C_T_* represents the difference between transcription of the tested gene and 16S rRNA gene in the same treatment group, and ΔΔ*C_T_* represents the difference between Δ*C_T_* (induced group) and the Δ*C_T_* (uninduced group). All samples were run in triplicate.

### Construction of promoter-*lacZ* transcriptional fusions

Two promoter-*lacZ* (*P_phbh1_* and *P_pcaA2_*) plasmids were constructed to identify inducible effectors by detecting *β*-galactosidase activity. The *P_phbh1_*-containing DNA fragment *PbR1* and the *P_pcaA2_*-containing DNA fragment *PbR2* were amplified from genomic DNA of strain H8 using the primer pairs pME-PbR1-F/pME-PbR1-R and pME-PbR2-F/pME-PbR2-R, respectively, as listed in Table 2. The PCR products were digested with EcoRI and PstI and then ligated into the EcoRI/PstI-digested pME6522 to generate reconstructed plasmids pME-*PbR1* and pME-*PbR2*. Subsequently, these plasmids were transformed into strains H8Δ*phbh2* and H8Δ*phbh1* to produce H8Δ*phbh2* (pME-*PbR1*) and H8Δ*phbh1* (pME-*PbR2*), respectively.

The cells of strains H8Δ*phbh2* (pME*-PbR1*) and H8Δ*phbh1* (pME*-PbR2*) were inoculated into YMS medium at an OD_600_ of 0.3. After adding 0.2 mM 3-Br-4-HB, Br-PCA, DBHB, or 4-HB, the cells were incubated at 37°C and 180 rpm for 3 h. The cells were then harvested by centrifugation and used for *β*-galactosidase activity analysis. *β*-galactosidase activity was measured using SDS- and chloroform-permeabilized cells as described (35, 52). All experiments were conducted in triplicate.

### Protein expression and purification

The *bhbR1* and *bhbR2* genes were amplified with primer pairs bhbR1-29f/r and bhbR2-29f/r, respectively, and then cloned into the pET29a(+) vector to generate expression plasmids pET-*bhbR1* and pET-*bhbR2*. The expression and purification of proteins with a C-terminal His tag were performed following a previously described method (32). The purified BhbR1 and BhbR2 were analyzed by SDS-PAGE and dialyzed in 3 L of PBS overnight to remove imidazole. Concentrations of purified proteins were determined using a BCA Protein Assay Kit (TaKaRa, Dalian, China) according to the manufacturer’s instructions.

### Gel filtration chromatography

Before gel filtration, purified BhbR1 and BhbR2 were concentrated using an ultrafiltration device (Amicon) with a 10-kDa cutoff membrane. To calibrate the molecular weights of BhbR1 and BhbR2, a panel of commercially available proteins, including myosin (200.0 kDa), phosphorylase B (97.2 kDa), serum albumin (66.4 kDa), egg albumin (44.3 kDa), papain (23.4 kDa), and lysozyme (14.7 kDa), was used as molecular weight markers. A solution containing BhbR1, BhbR2, or protein markers was charged onto a Superose 6 10/300 GL column (GE Healthcare) for gel filtration at 25°C using an ÄKTA Purifier chromatography system (GE Healthcare). The elution process was carried out with PBS at a flow rate of 0.4 mL min^−1^ for 1 h, with detection at a wavelength of 280 nm; elution volumes were systematically recorded.

### Electrophoretic mobility shift assay

The EMSA was conducted following previously described methods (32, 53). The DNA probes *PbR1* (a 243-bp DNA fragment containing *P_phbh1_* sequence) and *PbR2* (a 258-bp DNA fragment containing *P_pcaA2_* sequence) were amplified with primer pairs PbR1-f/r and PbR2-f/r, respectively. A non-specific 152-bp DNA probe amplified from the 16S rRNA gene of strain H8 using primer pairs Pb16S-f/r served as control DNA. The DNA probes (35 nM) were then incubated with increasing amounts of purified BhbR1 (0–0.6 µM) and BhbR2 (0–1.92 µM) in EMSA buffers. The buffer for BhbR1 contained 100 mM Tris–HCl (pH 8.0), 50 mM KCl, 5% (vol/vol) glycerol, and 1 mM dithiothreitol (DTT), while the buffer for BhbR2 contained 50 mM Tris-HCl (pH 8.0), 30 mM KCl, 5% (vol/vol) glycerol, 1 mM EDTA, and 5 mM DTT. After incubation at 25°C for 30 min, the resultant mixtures were immediately loaded onto a 5% native polyacrylamide gel at 4°C and electrophoresed at 100 V for 1.5 h using a 0.5× TGE buffer (45 mM Tris-boric acid and 1 mM EDTA, pH 8.0). The gels were subsequently stained with ethidium bromide for 10 min at room temperature to visualize DNA-protein interactions.

### DNase I footprinting assay

DNA probes *PbR1* and *PbR2* were cloned into pMD19-T (TaKaRa) to construct recombinant plasmids pMD-*PbR1* and pMD-*PbR2*, respectively. Subsequently, fluorescent 6-carboxyfluorescein (FAM)-labeled probes were generated by PCR amplification with primers FAM-M13F and M13R, using pMD-*PbR1* and pMD-*PbR2* as templates. For each DNase I footprinting assay, the binding reaction and sample analysis were conducted following a protocol described previously (32), modified by using the GeneScan-LIZ600 size standard from Applied Biosystems for sample analysis.

### SPR assay

SPR assays were performed to analyze intermolecular interactions using a Biomoleculer Interaction Analysis System (Biacore T200, Cytiva, Sweden) at room temperature. To determine the interaction between the regulator and the inducer, purified BhbR1 and BhbR2 were immobilized onto a CM7 sensor chip (Cytiva, Sweden) by amine coupling, achieving approximately 10,000 response units for each protein using the EDC/NHS method. A serial dilution of the inducer 3-Br-4-HB was prepared in PBST buffer (10 mM Na_2_HPO_4_, 2 mM KH_2_PO_4_, pH 7.4, 137 mM NaCl, 2.7 mM KCl, and 0.05% Tween 20) and was flowed across the chip at a rate of 30 µL min^−1^. The equilibrium dissociation constant (*K_D_*) was determined using the Biacore T200 Evaluation software with the steady-state affinity model.

To determine kinetic constants of regulator-promoter interactions, biotinylated DNA fragments *F_pbhb1_* (144 bp) and *F_pcaA2_* (153 bp) were generated by PCR amplification using primer pairs Fpb1-Btn-f/Fpb1-r and Fpb2-f/Fpb2-Btn-r, respectively. These biotinylated fragments were captured on a streptavidin sensor chip (Cytiva, Sweden) before introducing target proteins and monitoring interactions. As a control, a 152-bp nonspecific biotinylated DNA fragment derived from the 16S rRNA gene of strain H8 was amplified using primers F16S-Btn-f and F16S-r. Concentration gradients of BhbR1 and BhbR2 were prepared in HEPES-EP buffer (10 mM HEPES, 150 mM NaCl, 0.05% surfactant P20, and 3 mM EDTA, pH 7.4), with or without 5 µM 3-Br-4-HB; these were then applied to the chip. Kinetic analyses were conducted using the Biacore T200 Evaluation software with the kinetics affinity model.

### Chemical analysis

Qualitative and quantitative determination of 3-Br-4HB were carried out using a Dionex UltiMate 3000SD HPLC system equipped with a diode array detector and a Dionex C_18_ reversed-phase column (4.6 × 250 mm, 5-mm particle size), following the previously described method (21).

## Data Availability

The complete genome sequence of *Pigmentiphaga kullae* strain H8 has been deposited to the GenBank database under the accession number CP033966. The genes *bhbR1, orf404, pcaB, pcaA*, and *phbh1* in the *bhb1* cluster were assigned the locus tags EGT29_01975 to EGT29_01995 (bp 406719 to 411058). The genes *bhbR2, pcaA2, pcaB2*, and *phbh2* in the *bhb2* cluster were assigned the locus tags EGT29_16570 to 498 EGT29_16585 (bp 3505833 to 3508891).

## SUPPLEMENTAL MATERIAL

Supplemental file 1 aem.02403-24-s0001.doc Figures S1 to S5.

Supplemental file 2 aem.02403-24-s0002.docx Text S1, Table ST1, and Figure ST1.

ASM does not own the copyrights to Supplemental Material that may be linked to, or accessed through, an article. The authors have granted ASM a non-exclusive, world-wide license to publish the Supplemental Material files. Please contact the corresponding author directly for reuse.
