# Supplementary material for: Transcriptional regulation of two redundant 3-bromo-4-hydroxybenzoate catabolic operons via two different regulatory modes in Pigmentiphaga kullae strain H8
Source: Appl Environ Microbiol. 2025 Mar 4;91(4):e02403-24. doi: 10.1128/aem.02403-24 (PMC12016517; doi:10.1128/aem.02403-24)
Supplement: Supplemental file 1 — Figures S1 to S5. [file aem.02403-24-s0001.doc]

**Supplemental Material**

**Transcriptional regulation of two redundant 3-bromo-4-hydroxybenzoate catabolic operons via two different regulatory modes in *Pigmentiphaga kullae* strain H8**

Zhuang Ke ^1, 2, #^, Ke Yang ^1, #^, Zonghui Zhang ^1^, Ru Guo ^1^, Yuan Gao ^3^, Minjian Lan ^1^, Jiandong Jiang ^1^, Kai Chen ^1^, *

^1^ Department of Microbiology, College of Life Sciences, Nanjing Agricultural University, Key Laboratory of Agricultural and Environmental Microbiology, Ministry of Agriculture and Rural Affairs, Nanjing 210095, China.

^2^ College of Rural Revitalization, Jiangsu Open University, Nanjing 210036, China.

^3^ Central Laboratory of College of Horticulture, Nanjing Agricultural University, Nanjing 210095, China.

**^#^ Author Contributions**

Zhuang Ke and Ke Yang contributed equally to this paper.

* **Corresponding author**

Kai Chen

E-mail: chenkai@njau.edu.cn; Tel: 86-25-84399726

**Running title**: Regulation of 3-Br-4-HB catabolism by BhbR1 and BhbR2

**Key words:** LTTR; MarR family; transcriptional regulation; 3-bromo-4-hydroxybenzoate; *Pigmentiphaga* ***kullae***.

**
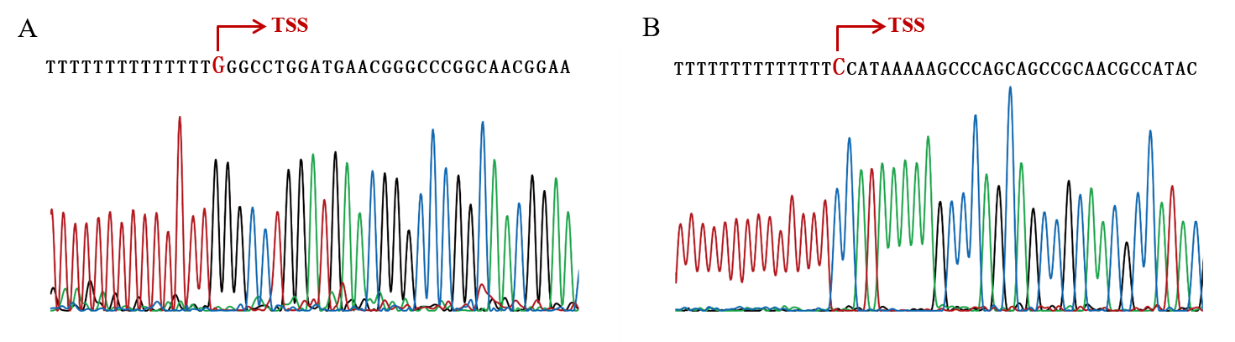
**

**Fig. S1.** Determination of transcriptional start sites (TSSs) of two catabolic operons by 5’-RACE. (A) The TSS of the operon *phbh1pcaApcaBorf404* was identified as a residue G, located 44 bp upstream of the translational start codon of *phbh1*. (B) The TSS of the operon *pcaA2pcaB2phbh2* was identified as a residue C, located 79 bp upstream of the translational start codon of *pcaA2*. The TSS residues are indicated by bent arrows, with the direction of the arrows indicating the direction of gene transcription.

**Fig. S2.** Prediction of Rho-dependent transcription terminators in operons *phbh1pcaApcaBorf404* (A) *and phbh2pcaB2pcaA2* (B) based on the RhoTherPredict algorithm. This algorithm essentially searches for a 78-nucleotide-long Rho utilization site (RUT site), characterized by a high C/G content and regularly spaced cytosine residues, followed by a putative pause site for RNA polymerase.


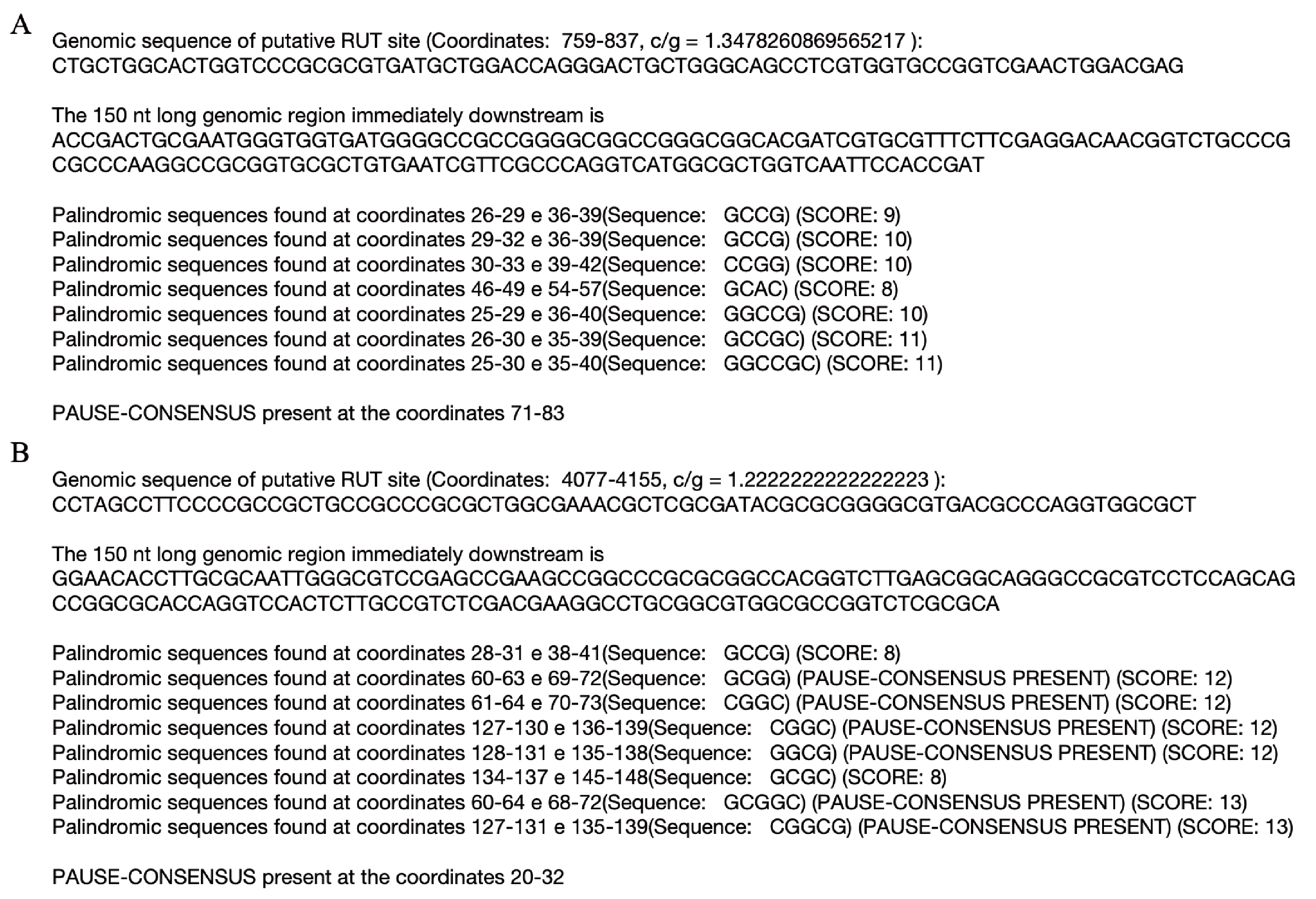


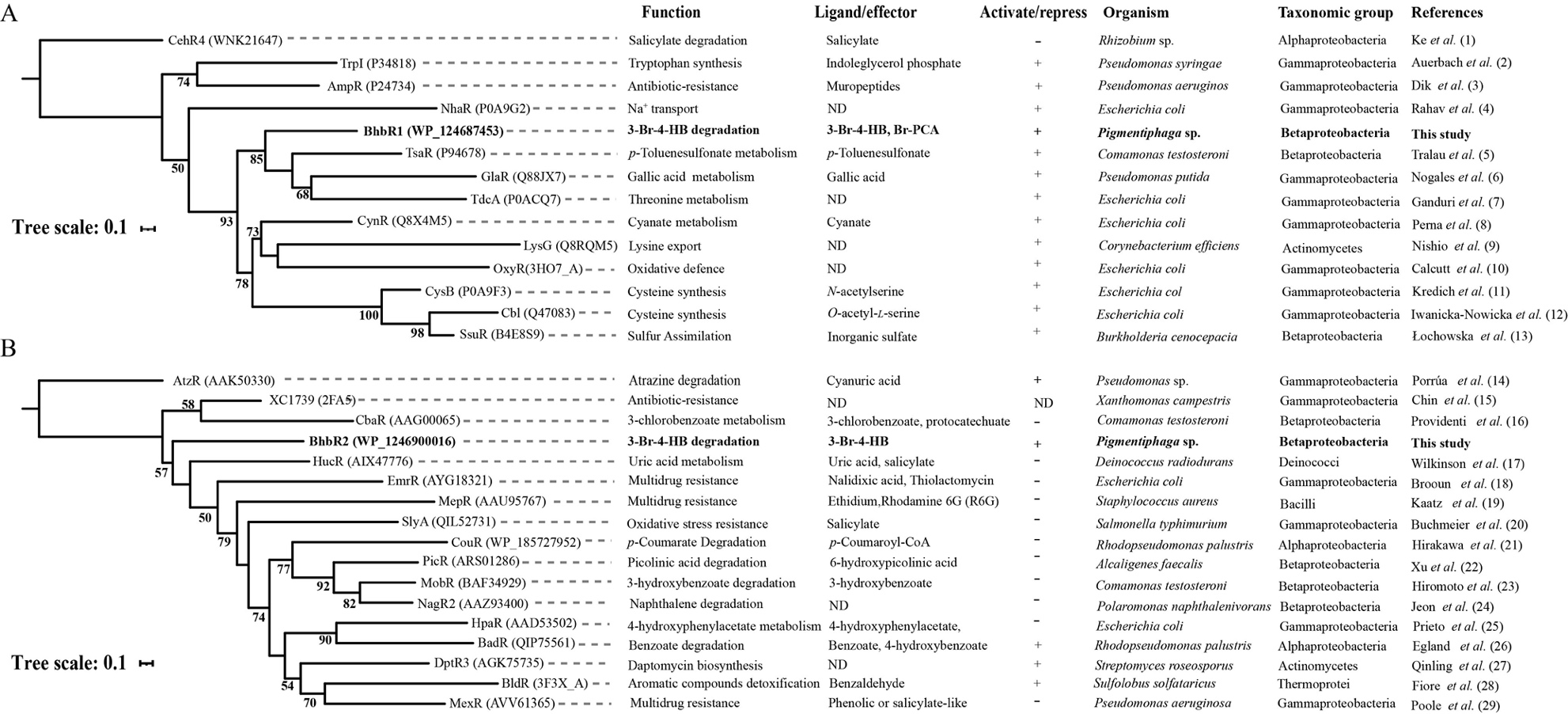


**Fig. S3.** Fig. S2. Phylogenetic analysis of BhbR1 and BhbR2. (A) The phylogenetic tree showing the relationship between BhbR1 and its closely related LysR-type transcriptional regulators (LTTRs). An IclR-type regulator, CehR4 (WNK21647), was used as the outgroup. (B) The phylogenetic tree depicting the relationship between BhbR2 and its related members of the MarR family. A LysR-type regulators AtzR (AAK50330) was used as the outgroup. Protein sequences were aligned with MAFFT L-INS-i v7.407, trimmed using TRIMAL 1.2rev59 (settings: -automated1). Phylogenetic trees were generated with IQ-TREE v2.1.3 using maximum likelihood (ML) methods and 1000 bootstrap replications. All trees were visualized by iTOL online software. Bootstrap values (above 50%) are indicated at tree nodes. Bar, 0.1 substitutions per amino acid position. Accession numbers of proteins from Non-redundant protein sequences, PDB and Swiss-port database were shown in parentheses. The regulated physiological and metabolic functions as well as the corresponding ligands/effectors are displayed after each protein. “+” indicates transcriptional activation; “−” represents transcriptional repression. 3-Br-4-HB, 3-bromo-4-hydroxybenzoate. Br-PCA, 3-bromo-4,5-dihydroxybenzoate.

**
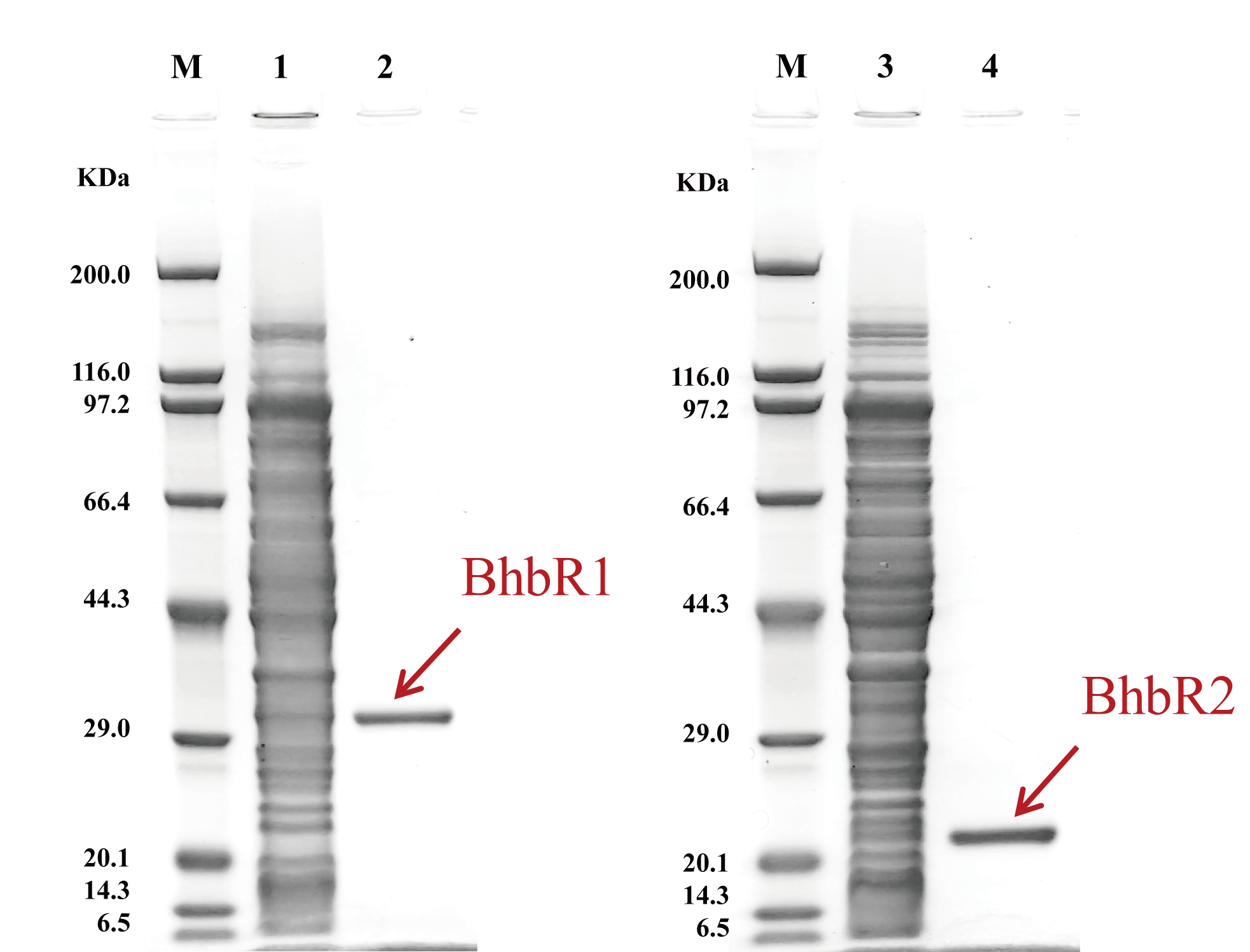
**

**Fig. S4.** SDS-PAGE of purified BhbR1 and BhbR2. Lane M, molecular weight markers; lanes 1 and 3, supernatants from induced cell lysates of *E. coli* BL21 (DE3) harboring pET-*bhbR1* and pET-*bhbR2*, respectively; lanes 2 and 4, purified BhbR1 and BhbR2, respectively, obtained through a Co^2+^-charged resin column.

**
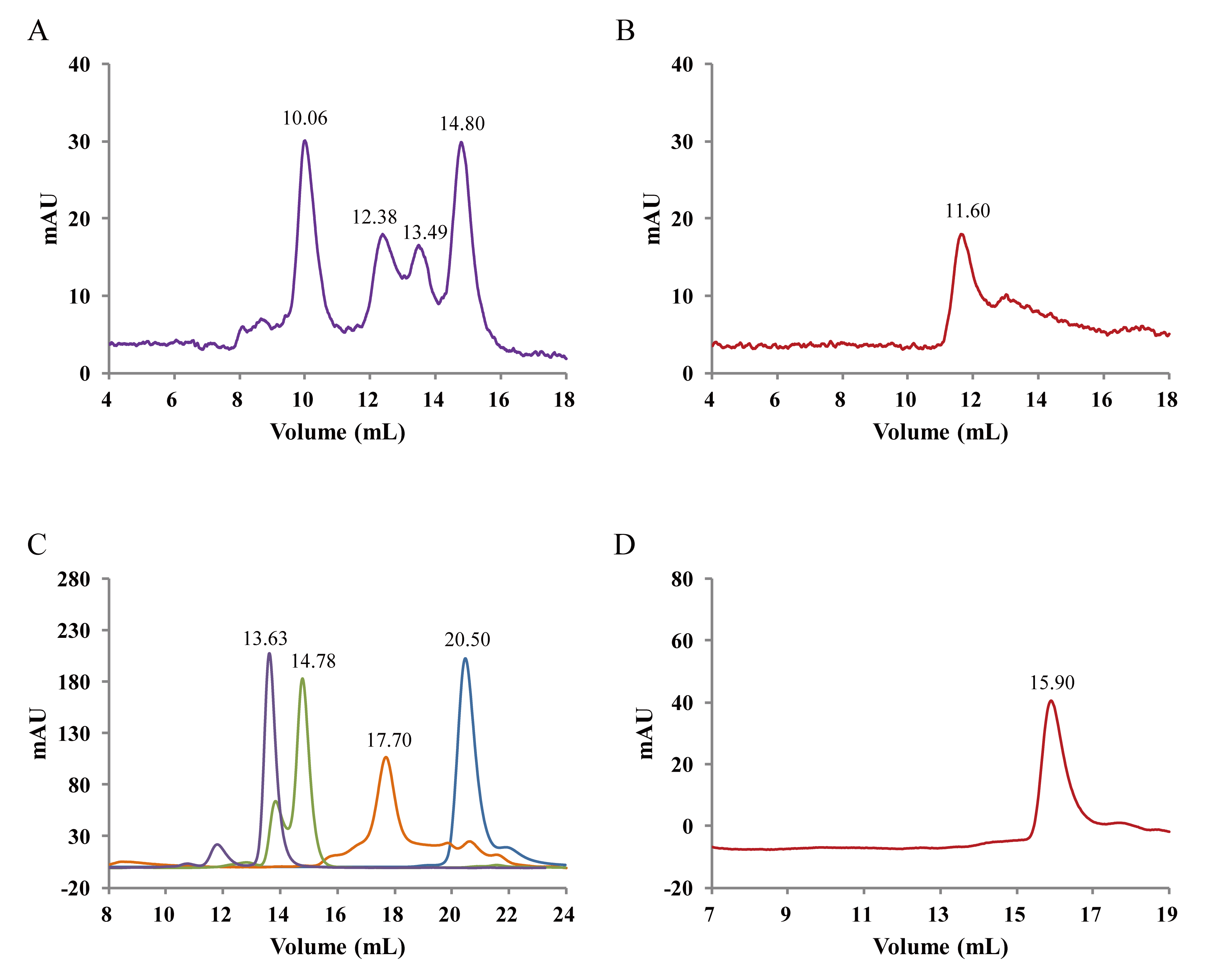
**

**Fig. S5.** Gel filtration of native BhbR1 and BhbR2. (A) Elution volumes of standard proteins: myosin (200.0 kDa, 10.06 mL), phosphorylase B (97.2 kDa, 12.38 mL), serum albumin (66.4 kDa, 13.49 mL), and egg albumin (44.3 kDa, 14.80 mL). (B) The elution volume of BhbR1 was 11.60 mL. The standard curve was fitted as y = -0.1386x + 3.6975 (R^2^ = 0.9997), where y strands for lg(Mw) and x for the elution volume. The native BhbR1 was calculated as 123.0 kDa. Since the theoretical Mw of monomeric BhbR1 is 33.4 kDa, native BhbR1 in solution is suspected to be a tetramer. (C) Elution volumes of standard proteins: serum albumin (66.4 kDa, 13.63 mL), egg albumin (44.3 kDa, 14.78 mL), papain (23.4 kDa, 17.70 mL), and lysozyme (14.7 kDa, 20.50 mL); (D) The elution volume of BhbR2 was 15.90 mL. The standard curve was fitted as y = -0.0934x + 3.0559 (R^2^ = 0.9836), where y strands for lg(Mw) and x for the elution volume. Native BhbR2 was calculated as 37.2 kDa. Since the theoretical Mw of monomeric BhbR2 is 20.9 kDa, native BhbR2 in solution is suspected to be a dimer.

**REFERENCES**

1. Ke Z, Zhu Q, Zhang M, Gao S, Jiang M, Zhou Y, Qiu J, Cheng M, Yan X, Wang J, Hong Q. 2023. Unveiling the regulatory mechanisms of salicylate degradation gene cluster cehGHIR4 in *Rhizobium* sp. strain X9. Appl Environ Microbiol 89(10):e0080223.
2. Auerbach S, Gao J, Gussin GN. 1993. Nucleotide sequences of the *trpI*, *trpB*, and *trpA* genes of *Pseudomonas syringae*: positive control unique to fluorescent pseudomonads. Gene 123(1):25-32.
3. Dik DA, Domínguez-Gil T, Lee M, Hesek D, Byun B, Fishovitz J, Boggess B, Hellman LM, Fisher JF, Hermoso JA, Mobashery S. 2017. Muropeptide Binding and the X-ray Structure of the Effector Domain of the Transcriptional Regulator AmpR of *Pseudomonas aeruginosa*. J Am Chem Soc 139(4):1448-1451.
4. Rahav-Manor O, Carmel O, Karpel R, Taglicht D, Glaser G, Schuldiner S, Padan E. 1992. NhaR, a protein homologous to a family of bacterial regulatory proteins (LysR), regulates *nhaA*, the sodium proton antiporter gene in *Escherichia coli*. J Biol Chem 267(15):10433-10438.
5. Tralau T, Mampel J, Cook AM, Ruff J. 2003. Characterization of TsaR, an oxygen-sensitive LysR-type regulator for the degradation of *p*-toluenesulfonate in *Comamonas testosteroni* T-2. Appl Environ Microbiol 69(4):2298-2305.
6. Nogales J, Canales A, Jiménez-Barbero J, Serra B, Pingarrón JM, García JL, Díaz E. 2011. Unravelling the gallic acid degradation pathway in bacteria: the gal cluster from *Pseudomonas putida*. Mol Microbiol 79(2):359-374.
7. Ganduri YL, Sadda SR, Datta MW, Jambukeswaran RK, Datta P. 1993. TdcA, a transcriptional activator of the *tdcABC* operon of *Escherichia coli*, is a member of the LysR family of proteins. Mol Gen Genet 240(3):395-402.
8. Perna NT, Plunkett G 3rd, Burland V, Mau B, Glasner JD, Rose DJ, Mayhew GF, Evans PS, Gregor J, Kirkpatrick HA, Pósfai G, Hackett J, Klink S, Boutin A, Shao Y, Miller L, Grotbeck EJ, Davis NW, Lim A, Dimalanta ET, Potamousis KD, Apodaca J, Anantharaman TS, Lin J, Yen G, Schwartz DC, Welch RA, Blattner FR. 2001. Genome sequence of enterohaemorrhagic *Escherichia coli* O157:H7. Nature 409(6819):529-533.
9. Nishio Y, Nakamura Y, Kawarabayasi Y, Usuda Y, Kimura E, Sugimoto S, Matsui K, Yamagishi A, Kikuchi H, Ikeo K, Gojobori T. 2003. Comparative complete genome sequence analysis of the amino acid replacements responsible for the thermostability of *Corynebacterium efficiens*. Genome Res 13(7):1572-1579.
10. Calcutt MJ, Lewis MS, Eisenstark A. 1998. The *oxyR* gene from *Erwinia carotovora*: cloning, sequence analysis and expression in *Escherichia coli*. FEMS Microbiol Lett 167(2):295-301.
11. Kredich NM. 1992. The molecular basis for positive regulation of cys promoters in *Salmonella typhimurium* and *Escherichia coli*. Mol Microbiol 6(19):2747-2753.
12. Iwanicka-Nowicka R, Hryniewicz MM. 1995. A new gene, *cbl*, encoding a member of the LysR family of transcriptional regulators belongs to *Escherichia coli* *cys* regulon. Gene 166(1):11-17.
13. Łochowska A, Iwanicka-Nowicka R, Zielak A, Modelewska A, Thomas MS, Hryniewicz MM. 2011. Regulation of sulfur assimilation pathways in *Burkholderia cenocepacia* through control of genes by the SsuR transcription factor. J Bacteriol 193(8):1843-1853.
14. Porrúa O, García-Jaramillo M, Santero E, Govantes F. 2007. The LysR-type regulator AtzR binding site: DNA sequences involved in activation, repression and cyanuric acid-dependent repositioning. Mol Microbiol 66(2):410-427.
15. Chin KH, Tu ZL, Li JN, Chou CC, Wang AH, Chou SH. 2006. The crystal structure of XC1739: a putative multiple antibiotic-resistance repressor (MarR) from *Xanthomonas campestris* at 1.8 A resolution. Proteins 65(1):239-242.
16. Providenti MA , Wyndham RC. 2001. Identification and Functional Characterization of CbaR, a MarR-Like Modulator of the *cbaABC*-Encoded Chlorobenzoate Catabolism Pathway. Appl Environ Microbiol 67(8):3530-3541.
17. Wilkinson SP, Grove A. 2004. HucR, a novel uric acid-responsive member of the MarR family of transcriptional regulators from *Deinococcus radiodurans*. J Biol Chem 279(49):51442-51450.
18. Brooun A, Tomashek JJ, Lewis K. 1999. Purification and ligand binding of EmrR, a regulator of a multidrug transporter. J Bacteriol 181(16):5131-5133.
19. Kaatz GW, DeMarco CE, Seo SM. 2006. MepR, a repressor of the *Staphylococcus aureus* MATE family multidrug efflux pump MepA, is a substrate-responsive regulatory protein. Antimicrob Agents Chemother 50(4):1276-1281.
20. Buchmeier N, Bossie S, Chen CY, Fang FC, Guiney DG, Libby SJ. 1997. SlyA, a transcriptional regulator of *Salmonella typhimurium*, is required for resistance to oxidative stress and is expressed in the intracellular environment of macrophages. Infect Immun 65(9):3725-3730.
21. Hirakawa H, Schaefer AL, Greenberg EP, Harwood CS. 2012. Anaerobic p-coumarate degradation by *Rhodopseudomonas palustris* and identification of CouR, a MarR repressor protein that binds *p*-coumaroyl coenzyme A. J Bacteriol 194(8):1960-1967.
22. Xu S, Wang X, Zhang F, Jiang Y, Zhang Y, Cheng M, Yan X, Hong Q, He J, Qiu J. 2022. PicR as a MarR Family Transcriptional Repressor Multiply Controls the Transcription of Picolinic Acid Degradation Gene Cluster pic in *Alcaligenes faecalis* JQ135. Appl Environ Microbiol 88(11):e0017222.
23. Hiromoto T, Matsue H, Yoshida M, Tanaka T, Higashibata H, Hosokawa K, Yamaguchi H, Fujiwara S. 2006. Characterization of MobR, the 3-hydroxybenzoate-responsive transcriptional regulator for the 3-hydroxybenzoate hydroxylase gene of *Comamonas testosteroni* KH122-3s. J Mol Biol 364(5):863-877.
24. Jeon CO, Park M, Ro HS, Park W, Madsen EL. 2006. The naphthalene catabolic (*nag*) genes of *Polaromonas naphthalenivorans* CJ2: evolutionary implications for two gene clusters and novel regulatory control. Appl Environ Microbiol 72(2):1086-1095
25. Prieto MA, Díaz E, García JL. 1996. Molecular characterization of the 4-hydroxyphenylacetate catabolic pathway of *Escherichia coli* W: engineering a mobile aromatic degradative cluster. J Bacteriol 178(1):111-120.
26. Egland PG, Harwood CS. 1999. BadR, a new MarR family member, regulates anaerobic benzoate degradation by *Rhodopseudomonas palustris* in concert with AadR, an *Fnr* family member. J Bacteriol 181(7):2102-2109.
27. Zhang Q, Chen Q, Zhuang S, Chen Z, Wen Y, Li J. A MarR Family Transcriptional Regulator, DptR3, Activates Daptomycin Biosynthesis and Morphological Differentiation in *Streptomyces roseosporus*. Appl Environ Microbiol 81(11):3753-3765.
28. Fiore A D , Fiorentino G, Vitale RM, Ronca R, Amodeo P, Pedone C, Bartolucci S, De Simone G. 2009. Structural analysis of BldR from *Sulfolobus solfataricus* provides insights into the molecular basis of transcriptional activation in Archaea by MarR family proteins. J Mol Biol 388(3):559-569.
29. Poole K, Tetro K, Zhao Q, Heinrichs DE, Bianco N. 1996. Expression of the multidrug resistance operon *mexA-mexB-oprM* in *Pseudomonas aeruginosa*: *mexR* encodes a regulator of operon expression. Antimicrob Agents Chemother 40(9):2021-2028.
30. Katoh K, Misawa K, Kuma KI, Miyata T. 2002. MAFFT: a novel method for rapid multiple sequence alignment based on fast Fourier transform. Nucleic Acids Res 30:059-3066.
31. Capella-Gutiérrez S, Silla-Martínez JM, Gabaldón T. 2009. trimAl: a tool for automated alignment trimming in large-scale phylogenetic analyses. Bioinformatics 25:1972-1973.
32. Letunic I, Bork P. 2019. Interactive Tree Of Life (iTOL) v4: recent updates and new developments. Nucleic Acids Res 47:W256-W259.
33. Minh BQ, Schmidt HA, Chernomor O, Schrempf D, Woodhams MD, Von Haeseler A, Lanfear R. 2020. IQ-TREE 2: new models and efficient methods for phylogenetic inference in the genomic era. Mol Biol Evol 37:1530-1534.
