# Supplementary material for: Transcriptional regulation of two redundant 3-bromo-4-hydroxybenzoate catabolic operons via two different regulatory modes in Pigmentiphaga kullae strain H8
Source: Appl Environ Microbiol. 2025 Mar 4;91(4):e02403-24. doi: 10.1128/aem.02403-24 (PMC12016517; doi:10.1128/aem.02403-24)
Supplement: Supplemental file 2 — Text S1, Table ST1, and Figure ST1. [file aem.02403-24-s0002.docx]

**Supplemental Text**

**Phylogenetic analysis of strain *Pigmentiphaga* sp. H8**

The 16S rRNA gene sequence of strain *Pigmentiphaga* sp. H8 was compared with sequences available in the NCBI GenBank database (www.ncbi.nlm.nih.gov/BLAST/) and EzBioCloud’s identification service (www.ezbiocloud.net/identify). To precisely define the taxonomic position of strain H8, a phylogenomic tree was constructed using its genome sequence of H8 and the maximum likelihood (ML) algorithm in IQ-Tree (1), with the best-fit model (LG+F+R4) and support values determined by 1,000 ultrafast bootstrap replicates. The digital DNA–DNA hybridization (dDDH) values between strain H8 and its phylogenetic neighbors were calculated using the Genome-to-Genome Distance Calculator (GGDC; http://ggdc.dsmz.de/distcalc2.php) according to the method of Meier-Kolthoff et al. (2).

As shown in Table ST1, the dDDH values between strain H8 and the related type strains of the genus *Pigmentiphaga* ranged from 14.10% to 74.90%. Notably, the dDDH value between strain H8 and *Pigmentiphaga kullae* K24^T^ was 74.90%, which exceeded the threshold value of 70% recommended for delineating prokaryotic species (2). This result suggests that strain H8 and *Pigmentiphaga kullae* K24^T^ belong to the same species within the genus Pigmentiphaga. Due to the limited number of strains within the genus *Pigmentiphaga* that have submitted their genome sequences to the NCBI database, a total of 6 strains’ genome sequences were retrieved. As shown in the Figure ST1, these 6 strains, along with strain H8, clustered together on the phylogenomic tree, distinguishing them from other strains of neighboring genera. Additionally, strain H8 formed a subclade with *Pigmentiphaga kullae* K24^T^, further supporting the conclusion that strain H8 belongs to the species *Pigmentiphaga kullae* within the genus *Pigmentiphaga.*

**REFERENCES**

1. Nguyen LT, Schmidt HA, von Haeseler A, Minh BQ. 2015. IQ-TREE: a fast and effective stochastic algorithm for estimating maximum-likelihood phylogenies. Mol Biol Evol 32:268-274.
2. Meier-Kolthoff JP, Auch AF, Klenk HP, Göker M. 2013. Genome sequence-based species delimitation with confidence intervals and improved distance functions. BMC Bioinformatics 14:60.

**Table ST1.** Results of genomic relatedness analyses based on the digital DNA-DNA hybridization (dDDH). The selection of reference strains is based on 16S rRNA gene identity. Strains to be compared: strain H8. Reference strains：1, *Pigmentiphaga kullae* K24^T^; 2, *Pigmentiphaga daeguensis* K110^T^; 3, *Pigmentiphaga humi* IMT-318^T^; 4, *Pigmentiphaga soli* BS12^T^; 5, *Pigmentiphaga litoral*is JSM061001^T^; 6, *Pigmentiphaga aceris* SAP-32^T^.

| **Reference strains** | **16S rRNA gene identity(%)** | | **dDDH (%)** | |
| --- | --- | --- | --- | --- |
| 1 | 99.72 | 74.90 | |  |
| 2 | 99.66 | 61.50 | |  |
| 3 | 98.60 | 27.30 | |  |
| 4 | 98.00 | 18.40 | |  |
| 5 | 97.86 | 16.20 | |  |
| 6 | 96.87 | 14.10 | |  |


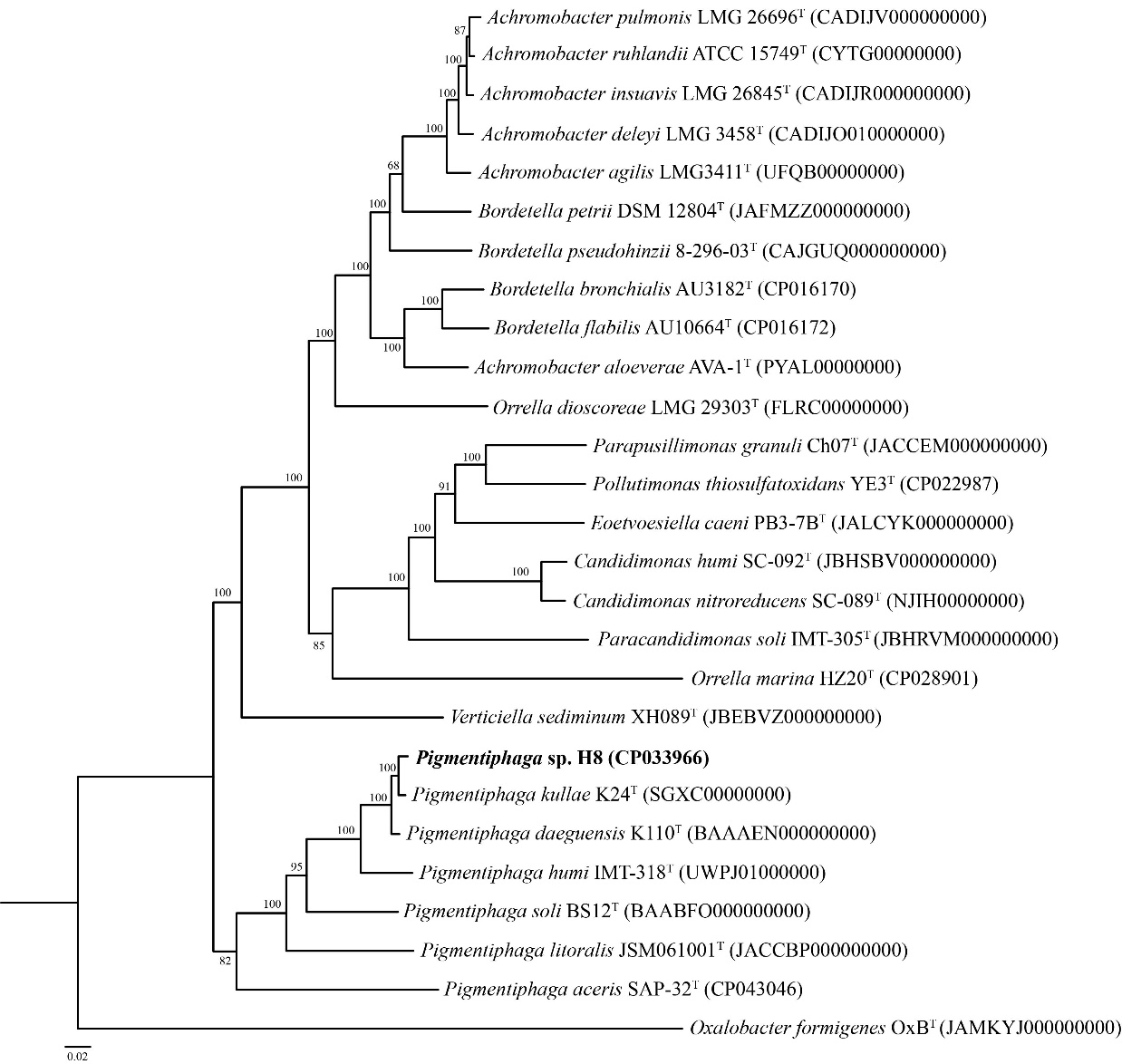


**Fig. ST1.** Phylogenomic tree based on genome sequences showing the phylogenetic position of strain H8 and closely related type strains in the genus *Pigmentiphaga* and related genera. *Oxalobacter formigenes* OxB^T^ was used as the outgroup. The tree was reconstructed using the ML algorithm. Genome accession numbers are indicated in parentheses. Bootstrap values based on 1000 replications are indicated at branch nodes. Bar, 0.02 substitutions per nucleotide position.
